# Iron Single‐Atom Catalyzed N‐Alkylation of Amines with Alcohols via Solvent‐Free Borrowing Hydrogen Strategy

**DOI:** 10.1002/advs.202507915

**Published:** 2025-08-19

**Authors:** Arun D. Kute, Hanumant B. Kale, Priti Sharma, Srinivasu Kancharlapalli, Gajanan Y. Shinde, Ruchi Chauhan, Ajay K. Singh, Shan Jiang, Jeffrey T. Miller, Radek Zboril, Yifeng Wang, Manoj B. Gawande

**Affiliations:** ^1^ Department of Industrial and Engineering Chemistry Institute of Chemical Technology Mumbai‐ Marathwada Campus Jalna Maharashtra 431213 India; ^2^ Regional Centre of Advanced Technologies and Materials Czech Advanced Technology and Research Institute Palacký University Olomouc 27 Šlechtitelů 779 00 Czech Republic; ^3^ Chemistry Division Bhabha Atomic Research Centre Mumbai 400085 India; ^4^ Department of Organic Synthesis and Process Chemistry CSIR‐Indian Institute of Chemical Technology Hyderabad 500007 India; ^5^ Davidson School of Chemical Engineering Purdue University 480 Stadium Mall Dirve West Lafayette IN 47906 United States; ^6^ Nanotechnology Centre Centre for Energy and Environmental Technologies VSB–Technical University of Ostrava 17. Listopadu 2172/15 Ostrava‐Poruba 708 00 Czech Republic; ^7^ Department of Chemistry and Chemical Engineering Shandong University Shandong P. R. China

**Keywords:** borrowing hydrogen, flow chemistry, iron, microwave‐assisted, n‐alkylation, single‐atom catalyst

## Abstract

Industrial hydrogenation is a pivotal process in chemical synthesis. However, it has significant drawbacks, including high cost, safety risks associated with the use of molecular hydrogen gas, and substantial energy demands due to the need for elevated temperatures and pressures to achieve satisfactory yields. The borrowing hydrogen synthesis, which enables the transfer of hydrogen between molecules, offers a promising approach for green, one‐pot synthesis of industrially important chemicals and intermediates. Despite its potential, the broad application remains limited due to the reliance on toxic solvents, expensive noble metal catalysts, and the still restricted efficiency and substrate scope. In this study, the first solvent‐free strategy for the *N*‐alkylation of amines with alcohols is presented, employing an N‐doped graphene‐supported Fe single‐atom catalyst (Fe_SA_@N‐G; 1.06 wt.%). This approach achieves superior conversion and selectivity (up to 99%) along with record values for turnover number (TON, 1032.7) and turnover frequency (TOF, 413.1 h^-1^) for the coupling reaction of aniline with benzyl alcohol, surpassing all previously reported catalysts. DFT calculations, combined with experimental data, elucidated the reaction mechanism and identified the Fe_1_(III)‐N_4_ active site participating in Fe‐H hydride transfer and containing two pyrrolic and two pyridinic nitrogens bound to the Fe center. The developed technology is further supported by the catalyst's excellent scalability, reusability, and performance under continuous‐flow conditions. Additionally, the exceptional efficiency of the single‐atom catalyst is demonstrated across more than 50 substrates, including reactions involving both aliphatic and aromatic amines with aliphatic and aromatic alcohols. The industrial applicability of this technology is validated through the synthesis of pharmaceutically relevant compounds, including stimulant drugs, antihistamines, and pharmaceutical intermediates.

## Graphical abstract

An innovative iron‐based single‐atom catalyst (Fe_SA_@N‐G) with Fe_1_–N_4_ coordination is presented, synthesized through a simple and scalable method. It exhibits exceptional activity and selectivity in N‐alkylation via the borrowing hydrogen strategy under solvent‐free conditions, producing water as the only by‐product. Achieving record TOF (413.1 h^-1^) and TON (1032.7), it surpasses all reported noble and non‐noble metal heterogeneous catalysts.

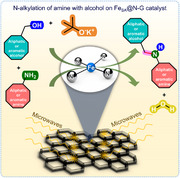

## 1 Introduction

In the realm of chemistry, hydrogenation is recognized as a pivotal process for the synthesis of a broad range of crucial chemicals.^[^ ^1^, ^2^, ^3^ ^]^ It is widely utilized for the synthesis of fine chemicals due to its versatility in producing a wide range of products.^[^ ^4^, ^5^ ^]^ However, it suffers from several drawbacks, including high cost of investment in terms of closed reactor systems, safety risks due to the use of molecular hydrogen gas, and a huge energy demand related to the high temperature and pressure required to achieve adequate yields.

Transfer hydrogenation, or hydrogen‐borrowing, offers a sustainable and environmentally benign option for hydrogenation reactions by allowing the transfer of hydrogen between molecules, eliminating the need for direct use of hydrogen gas.^[^ ^6^ ^]^ The hydrogen‐borrowing method involves the back‐and‐forth transfer of hydrogen. Among various chemicals, the alcohols are inexpensive and environmentally friendly alkylating reagents, which can be used in the synthesis of compounds using the borrowed hydrogen methodology.^[^ ^7^ ^]^

Nitrogen‐containing organic molecules are essential to synthetic organic chemistry and basic building blocks of naturally occurring, biologically active compounds. Among *N*‐bearing organic compounds, *N*‐substituted amines play a crucial role in various industries such as pharmaceuticals, agrochemicals, dyes, and surfactants.^[^ ^8^ ^]^

The utilization of alcohols as the alkylation reagent in the synthesis of N‐alkylated amines offers a solution to avoid the use of risky oxidizing or reducing reagents and the production of salt waste.^[^ ^28^, ^29^ ^]^ The *N*‐alkylation of amines with alcohols reaction produces amines in higher order.^[^ ^30^ ^]^ It serves as an attractive and environmentally sustainable choice in contrast to traditional methods such as alkylating amines with alkyl halides or reducing carbonyl compounds through reductive amination.^[^ ^31^, ^32^ ^]^ In the borrowing hydrogen (BH) *N*‐alkylation process, an alcohol is first turned into a reactive carbonyl molecule by oxidation with the help of a catalyst. Before the catalyst regenerates itself by returning the “borrowed” hydrogen, this intermediate may undergo several other reactions.

The use of BH or hydrogen auto‐transfer (HAT) methodology in alcohol amination generally provides the following benefits: a) The utilization of alcohols as readily available and cost‐effective starting materials, including bio‐based feedstocks; b) The alcohol acts as its own hydrogen donor, eliminating the need for external hydrogen; and c) water being the only byproduct makes the process sustainable and environmentally friendly. Thus, *N*‐alkylation of amines with alcohols via the borrowing hydrogen strategy holds significant value in the field of organic synthesis and has made considerable progress in green chemistry due to its ability to achieve high atom efficiency and eliminate the formation of solid byproducts.^[^ ^33^ ^]^

**Figure 1:**
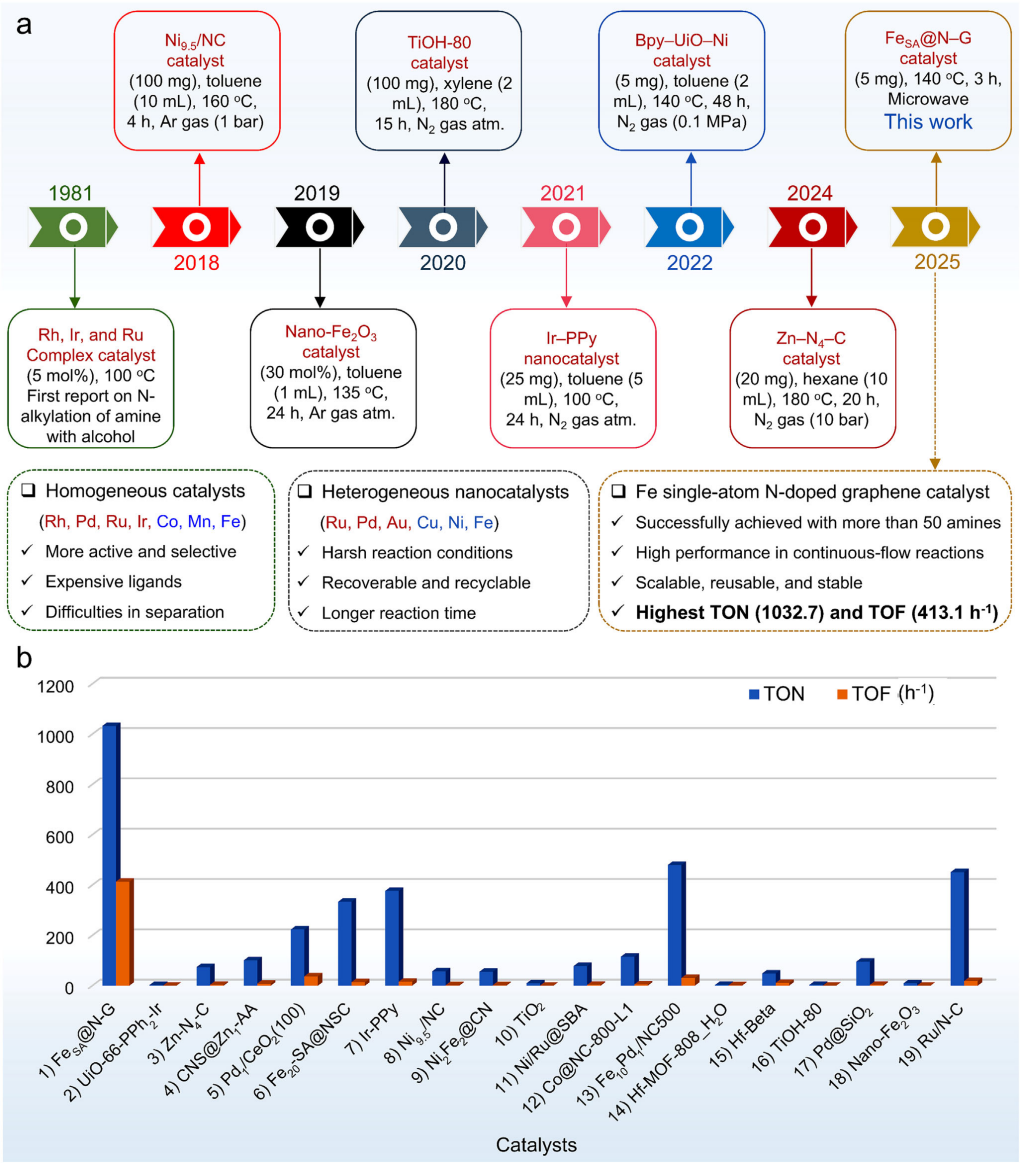
a) Pictorial overview of heterogeneous catalysts reported for *N*‐alkylation reaction. (Ni_9.5_/NC‐Single Ni@N_4_ sites in nitrogen‐doped carbon;^[^ ^9^ ^]^ Nano‐Fe_2_O_3_–Commercial nano‐Fe_2_O_3_;^[^ ^10^ ^]^ TiOH‐80 – Titanium hydroxide;^[^ ^11^ ^]^ Ir‐PPy NPs–Ir single‐atom doped polypyrrole nanoparticles;^[^ ^12^ ^]^ Bpy‐UiO‐Ni – Ni(II)‐bipyridine‐based metal‐organic framework;^[^ ^13^ ^]^ Zn–N_4_–C N‐doped carbon supported zinc single‐atom.^[^ ^14^ ^]^ b) Comparison of TON and TOF values of recently reported heterogeneous catalysts for the coupling of aniline with benzyl alcohol reaction (Catalysts: 1‐This work, 2,^[^ ^15^ ^]^ 3,^[^ ^14^ ^]^ 4,^[^ ^16^ ^]^ 5^[^ ^17^ ^]^ 6,^[^ ^18^ ^]^ 7,^[^ ^12^ ^]^ 8,^[^ ^9^ ^]^ 9,^[^ ^19^ ^]^ 10,^[^ ^20^ ^]^ 11,^[^ ^21^ ^]^ 12,^[^ ^22^ ^]^ 13,^[^ ^23^ ^]^ 14,^[^ ^24^ ^]^ 15,^[^ ^25^ ^]^ 16, ^11^ ^]^ 17,^[^ ^26^ ^]^ 18^[^ ^10^ ^]^ and 19^[^ ^27^ ^]^).

In 1981, Grigg and Watanabe pioneered the exploration of the BH methodology for *N*‐alkylation reactions^[^ ^34^, ^35^ ^]^ see **Figure** **1**a for a historical overview. Since this first report, various homogeneous as well as heterogeneous noble and non‐noble metal catalysts have been employed for the *N*‐alkylation reaction.^[^ ^36^, ^37^, ^38^, ^39^, ^40^, ^41^, ^42^, ^43^, ^44^, ^45^, ^46^, ^47^, ^48^, ^49^, ^50^ ^]^ However, several challenges persist, including high cost, high catalyst loading, low product selectivity, and the requirement for high pressure, inert atmosphere, high temperature, or cocatalysts.

Recent developments in the BH strategy emphasize the utility of reusable 3d transition metal catalysts, such as Fe, Mn, Co, Ni, and Cu, as sustainable alternatives to precious metals.^[^ ^33^ ^]^ These earth‐abundant metals offer cost‐effectiveness, low toxicity, and promising activity for C–N coupling reactions, demonstrating significant catalytic activity through the BH strategy. The first report on single‐atom catalysts (SACs), featuring Ni‐NC SAC for effective *N*‐alkylation of amines with alcohols, was reported by Su et al.^[^ ^9^ ^]^ However, high loadings of catalyst and high reaction temperatures, e.g., 160 °C, were required for the *N*‐alkylation reaction. Additionally, this Ni‐NC SAC catalyst was unsuccessful in the *N*‐alkylation of aniline with aliphatic alcohols.^[^ ^9^ ^]^ Among iron‐based heterogeneous catalysts, a selective Fe_3_O_4_ oxide nanocatalyst was reported for the *N‐*alkylation of amines using alcohols via hydrogen auto‐transfer synthesis.^[^ ^51^ ^]^ However, the slow reaction kinetics (7 days), limited substrate scope, toxic solvent, and excess amount of base are the main drawbacks of this process. Similarly, Namitharan and co‐workers reported higher yields for the *N‐*alkylation of amines with alcohol catalyzed by nano‐Fe_3_O_4_;^[^ ^10^ ^]^ however, large amounts of catalyst (i.e., 30 mol%), long reaction times, and organic solvent were necessary to achieve a good conversion. A single‐atom Fe_20_‐SA@NC catalyst was also demonstrated for the purpose of *N*‐alkylation reaction. However, this catalyst was ineffective at *N*‐alkylation with aliphatic alcohols, which required long reaction times and resulted in low TON and TOF values.^[^ ^18^ ^]^

Further developed Ir, Zn, Ni‐based single‐atom materials exhibited good conversion but low product selectivity, and required harsh reaction conditions, as well as longer reaction time.^[^ ^12^, ^13^, ^16^ ^]^ Moreover, Lui et al. reported a single‐atom catalyst based on the typically inert Zn–N_4_–C structure derived from ZIF‐8, demonstrating significant catalytic activity in alcohol activation, thus facilitating alcohol‐mediated alkylation through auto transfer hydrogenation.^[^ ^14^ ^]^ However, the earlier protocol stipulated a reaction under more rigorous conditions N_2_ (10 bar), 180 °C temperature, and prolonged reaction time. Very recently, Wendt and co‐workers reported 25 mol% base‐catalyzed *N*‐alkylation of amines with alcohols; however, *t*‐BuOK fails to convert aliphatic, cyclic, and electron withdrawing substituted alcohols.^[^ ^52^ ^]^

From the perspective of sustainable and green chemistry, future developments in *N*‐alkylation methods for amine synthesis will increasingly focus on robust and cost‐effective reusable 3d transition metal catalysts, operating under mild and environmentally friendly conditions. Among these 3d transition metals, iron‐based catalysts, especially those anchored on nitrogen‐doped graphene in the form of single atoms, will continue to gain prominence in the BH strategy due to their potential for high activity, selectivity, and long‐term stability. This includes the redox behavior and strong metal support coordination of Fe‐N in the catalytic system. These catalysts will offer atomically dispersed active sites and strong metal–support interactions, which will enhance catalytic efficiency, and provide high TON, and TOF.

Herein, we developed a reusable and recyclable N‐doped graphene‐supported iron single‐atom (Fe_SA_@N‐G) via an ion adsorption strategy for the microwave‐assisted *N*‐alkylation of amines with alcohols. Experimental results demonstrate that the Fe_SA_@N‐G catalyst exhibits outstanding catalytic performance with excellent product selectivity across a range of substrates, including aliphatic, cyclic, and electron‐withdrawing substituted amines with alcohols. Notably, this catalytic protocol achieves significantly high TOF and TON values (Figure 1b), compared to most previously reported noble and non‐noble metal nanocatalysts and SACs.(Table S1, Supporting Information).

Importantly, the developed mixed valence state iron single‐atom catalyst with Fe‐N_4_ coordination works without the use of any solvent. Moreover, this first solvent‐free strategy allows working with a very low amount of catalyst, i.e., 0.053 wt.% of Fe loading, a short reaction time, and moderate reaction temperature, thus having the potential of an immediate impact in industrial practice.

## 2 Results and Discussion

### 2.1 Catalysts Synthesis and Characterization

**Figure 2:**
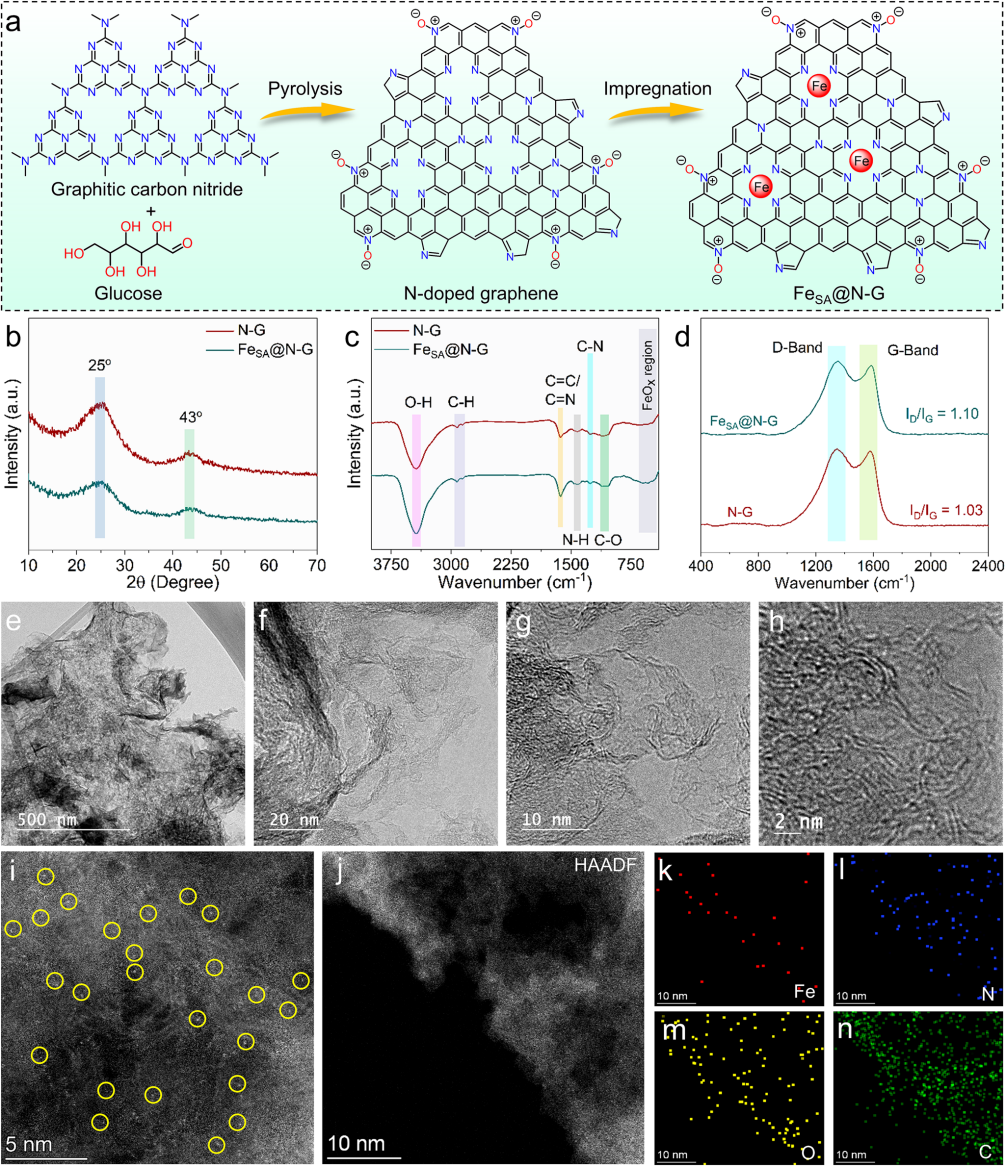
a) Schematic representation of the fabrication process for Fe_SA_@N‐G catalyst, b) XRD pattern of N‐G, and Fe_SA_@N‐G, c) FTIR spectra of N‐G and Fe_SA_@N‐G, d) Raman spectra of N‐G and Fe_SA_@N‐G. Microscopic characterization of Fe_SA_@N‐G catalyst: e‐h) HR‐TEM images, i) HAADF‐STEM image (Fe single atoms are highlighted in yellow circles), j) HAADF‐STEM image, and k‐n) elemental mapping of Fe, N, O, and C, respectively.

The synthesis of Fe_SA_@N‐G was achieved through an ion adsorption and microwave heating approach, illustrated schematically in **Figure** **2**a.

In particular, graphitic carbon nitride (g‐C_3_N_4_) and glucose were first assembled through high‐temperature pyrolysis to produce N‐doped graphene (N‐G) as a support for anchoring Fe single atoms. Then, an aqueous solution of iron(III) nitrate salt was added dropwise under sonication. Finally, sodium borohydride was added, and the dispersion was irradiated with microwaves before freeze‐drying. For comparative purposes, the synthesis of Fe_NP_@N‐G catalyst, containing iron nanoparticles (NPs) embedded in the N‐G support, was simply achieved by omitting the microwave treatment (for a detailed procedure, see Experimental section 2 and Characterization in Supporting Information). A large set of characterization techniques was used to gain insight into the chemical, structural, and electronic features of Fe_SA_@N‐G catalyst, including comparative analyses with properties of the N‐G support and Fe_NP_@N‐G catalyst.

XRD patterns of the N‐G support and Fe_SA_@N‐G catalyst revealed the presence of two broad peaks at ≈25° and 43°, which can be attributed to the (002) and (101) planes of N‐doped graphene or graphitic carbon materials (Figure 2b).^[^ ^53^ ^]^ No peaks corresponding to Fe or Fe─O based NPs were observed in the Fe_SA_@N‐G pattern (Figure 2b).^[^ ^23^ ^]^ Fourier transform infrared (FT‐IR) spectra show distinct stretching vibration peaks at ≈3437 (O─H), ≈2925 (C─H), ≈1628 (C═N/C═C), ≈1418 (N─H), ≈1258 (C─N), and ≈1105 cm^−1^ (C─O), which represent the main chemical groups involved in the structure of the N‐G support. The vibration peak corresponding to Fe─O bonding was not observed in the FT‐IR pattern of Fe_SA_@N‐G (Figure 2c). Thus, the XRD pattern and FT‐IR spectrum of Fe_SA_@N‐G confirmed that the microwave treatment prevented the agglomeration of iron species, enabling the embedding of iron single atoms in the structure of N‐G support. The Raman spectra of N‐G and Fe_SA_@N‐G display two distinct peaks at ≈1329 and 1586 cm^−1^, which correspond to the clearly defined D band and G band in the graphene structure, respectively. The I_D_/I_G_ ratio of Fe_SA_@N‐G is 1.10, which is higher than that of the pristine N‐G support (1.03), reflecting the defective character of the Fe_SA_@N‐G structure due to the microwave treatment (Figure 2d).^[^ ^54^, ^55^ ^]^

High‐resolution transmission electron microscopy (HR‐TEM) images (Figure 2e–g) of Fe_SA_@N‐G catalyst revealed transparent sheets with highly wrinkled morphology. No Fe/Fe─O based NPs were observed in the microwave‐treated Fe_SA_@N‐G catalyst (Figure 2h). In addition, high‐angle annular dark‐field scanning transmission electron microscopy (HAADF‐STEM) was employed for the confirmation of the presence of Fe single atoms. The HAADF‐STEM image exhibited distinct bright spots corresponding to individual Fe atoms (indicated by the yellow circles) on the N‐G support. This observation suggests that Fe_SA_@N‐G possesses a high concentration of atomic Fe sites dispersed over the N‐G matrix (Figure 2i). The HADDF‐STEM image, along with the accompanying energy‐dispersive X‐ray (EDX) mappings, demonstrated the uniform distribution of Fe single atoms within the N‐G support (Figure 2j–n).

**Figure 3:**
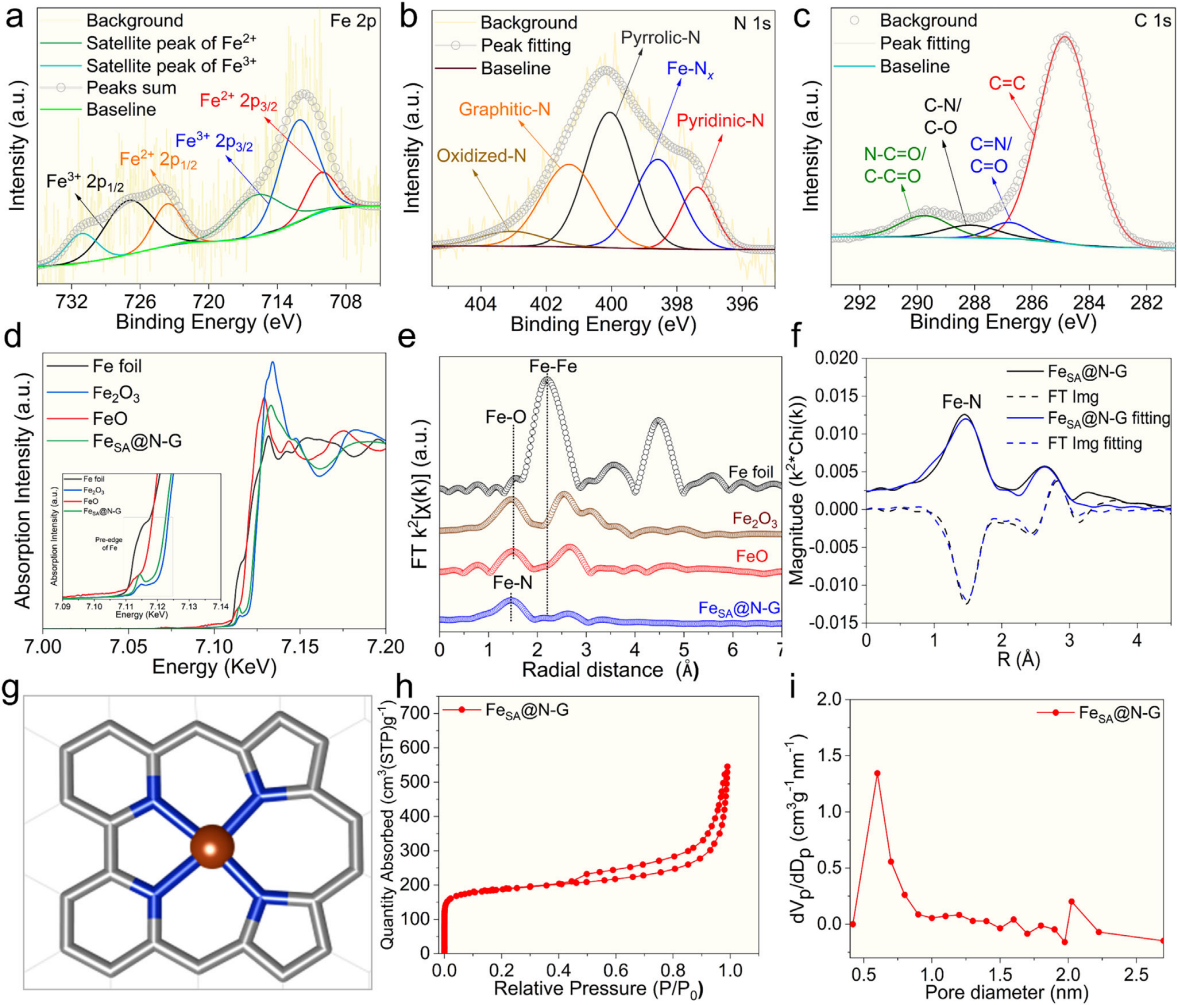
High resolution XPS spectra of Fe_SA_@N‐G catalyst: a) Fe 2p deconvolution, b) N 1s deconvolution, and c) C 1s deconvolution, d) Fe K‐edge XANES spectra, e) Fourier transform of EXAFS spectra, f) EXAFS curve fitting of Fe_SA_@N‐G catalyst, g) 3D model of Fe_SA_@N‐G, h) Nitrogen (N_2_) adsorption–desorption isotherms of Fe_SA_@N‐G, i) Pore size distribution in Fe_SA_@N‐G structure as derived from N_2_ adsorption‐desorption curves.

The elemental composition and surface structure of the Fe_SA_@N‐G catalyst were determined using X‐ray photoelectron spectroscopy (XPS) measurements. The XPS survey scan demonstrated the presence of Fe, C, N, and O elements in Fe_SA_@N‐G (Figure S6a and Table S2, Supporting Information). In the high‐resolution XPS Fe 2p spectrum of Fe_SA_@N‐G, the presence of distinct peaks with binding energies of 712.16 and 727.03 eV suggests a higher oxidation state of Fe^3+^, contrasting with the peaks at 710.19 and 723.63 eV, which indicate the Fe^2+^ (**Figure** **3**a).^[^ ^56^, ^57^ ^]^ The deconvolution of high‐resolution N 1s spectrum revealed the coexistence of pyridinic‐N (397.38 eV), Fe‐N_x_ (398.58 eV), pyrrolic‐N (400.05 eV), graphitic‐N (401.29 eV), and oxidized‐nitrogen species (403.06 eV). The presence of the Fe‐N_x_ peak signifies that iron single atomic species were coordinated with nitrogen atoms in the structure of the N‐G support (Figure 3b).^[^ ^58^ ^]^ The deconvolution of C 1s XPS spectrum revealed four distinct peaks, including 284.84, 286.79, 288.14, and 289.75 eV, which can be assigned to C═C (sp^2^), C═O/C═N, C─O/C─N, and N─C═O/C─C═O, respectively (Figure 3c).^[^ ^58^ ^]^

To bring a deeper insight into the electronic properties and local coordination of Fe single atoms, the Fe_SA_@N‐G catalyst was characterized by X‐ray absorption near edge spectroscopy (XANES) and extended X‐ray absorption fine structure (EXAFS) methods. By analyzing the XANES spectra, it is evident that the rising‐edge position of Fe_SA_@N‐G lies between the corresponding Fe foil and iron oxides (Figure 3d). The energy of the pre‐edge peak in the XANES spectrum is a 1s to 3d dipole forbidden transition, and the energy is sensitive to the oxidation state. The pre‐edge XANES peak of Fe_SA_@N‐G is 7.1144 keV (Table S4, Supporting Information), and is identical to that of Fe_2_O_3,_ indicating that the majority of Fe ions are in a 3+ oxidation state.^[^ ^58^ ^]^ The local Fe^3+^ coordination geometry was determined by fitting the k^2^‐weighted fourier transform of EXAFS (Figure 3e). The Fe_SA_@N‐G exhibits a singular primary peak at ≈1.49 Å (phase uncorrected distance), which was fitted with a Fe‐N scattering path of 3.8 Fe‐N bonds at 2.00 Å (Table S4, Supporting Information). This clearly indicates that Fe(III) and Fe(II) single atoms are four‐coordinated in planar Fe‐N_4_ geometry.^[^ ^54^ ^]^

Additionally, there are no peaks in the EXAFS spectrum, which would be assigned to Fe NPs at 2.17 Å. However, a small higher shell peak is present at 2.6 Å, which indicates a small fraction of Fe oxide NPs in the sample. In summary, X‐ray absorption spectroscopy (XAS) spectra of Fe_SA_@N‐G are consistent with the majority of Fe atoms present as isolated 3+ ions coordinated to four nitrogens from the N‐G support at 2.00 Å (Figure 3f; Table S4, Supporting Information). The model image of Fe(III) atoms coordinated with four nitrogens (2 pyridinic‐N and 2 pyrrolic‐N) (Fe‐N_4_) is depicted in (Figure 3g). The porosity and Brunauer‐Emmett‐Teller (BET) surface area of Fe_SA_@N‐G were quantified through the measurements of N_2_ adsorption‐desorption isotherms (Figure 3h). The Fe_SA_@N‐G material exhibits a large surface area of 788 m^2^/g and dominantly microporous character with the maximum of the size distribution at 0.6 nm (Figure 3i; Table S5, Supporting Information).

Finally, Fe content in Fe_SA_@N‐G was determined to be 1.06 wt.% via inductively coupled plasma mass spectroscopy (ICP‐MS). Thus, we can conclude that Fe_SA_@N‐G catalyst contains mixed valence Fe(II)/Fe(III) single atoms in Fe_1_‐N_4_ tetrahedral coordination. The iron single atoms are embedded in microporous N‐G support in a concentration of ca 1.0 wt.%.

### 2.2 Optimization of Reaction Conditions for N‐Alkylation of Amines with Alcohol

The developed catalytic materials were tested in the model reaction of aniline (1a) with *n*‐octanol (2a) to obtain *N*‐octylaniline product (3a) (**Table** **1**) under various conditions, including catalyst quantity, different bases, reaction temperature, and the use/absence of the microwave (MW) reactor. The desired product (entry 1) was not achieved in the blank run, which was conducted without any catalyst or base. The sole potassium *tert*‐butoxide (*t*‐BuOK) base‐catalyzed reaction yielded inadequate conversion (36%) toward the desired product (entry 2). Notably, without a base, the Fe_SA_@N‐G catalyst failed to proceed with this transformation (entry 3). Moreover, the utilization of the Fe_SA_@N‐G catalyst with a *t*‐BuOK exhibited exceptional conversion (88%) and selectivity (99%) to the desired product (3a) (entry 4). However, the control catalysts, such as FeCl_3_, N‐G, and Fe_NP_@N‐G, when explored with a base, did not yield a satisfactory amount of the product, providing 67%, 40%, and 60% amine conversion, respectively (entries 5, 6, and 7). Based on the findings from previous entries 2, 4, 5, 6, and 7, it can be inferred that the Fe_SA_@N‐G catalyst exhibits enhanced catalytic activity, facilitating the transfer of borrowing hydrogen to the desired *N*‐alkylated product. Despite the increasing quantity of catalyst (5 vs 10 mg), there is no improvement in conversion to achieve selective product within the same reaction time (entry 8). The increase in the reaction time 3 to 4 h did not affect the conversion (88%) toward the *N*‐alkylated product (3a) (entry 10), while a decreased reaction time (2 h) caused the drop in conversion to 59% (entry 9). The *N*‐alkylated product (3a) was obtained at 90% at 160 °C, while a drastic decrease in conversion (17%) was observed at a lower temperature, i.e., 120 °C, respectively (entries 11 and 12). Interestingly, altering the base concentration from 0.25 mmol to 0.75 mmol led to increased conversion from 76% up to 90% (entries 13 and 14). The reaction was also examined at varying concentrations of *n*‐octanol as starting materials to obtain the desired product (90%) (entry 15). Moreover, the utilization of other bases such as KOH and NaOH during the reaction resulted in noticeably lower yields of the *N*‐alkylated product, i.e., 65% and 60%, respectively (entries 16 and 17). The utilization of solely thermal conditions (without microwave assistance), particularly at 140 °C for 3 h, led to a substantial decline in amine conversion (13%) in comparison with microwave‐assisted *N*‐alkylation reaction (entry 18). The effectiveness of the MW reactor in activating Fe_SA_@N‐G catalysts and increasing the rate of reaction for the *N*‐alkylation of aniline with *n*‐octanol is evident in entry 4, representing optimum reaction conditions for the model reaction.

**Table 1:** The investigation of different reaction conditions for the microwave‐assisted *N*‐alkylation of aniline with *n*‐octanol.^a)^

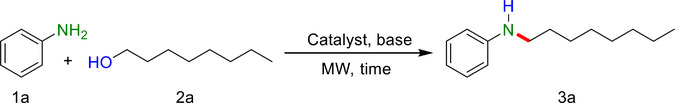			
Entry No.	Catalyst	1a Conv. (%)	3a Sel. (%)
1	−	0	0
2	−	36	99
3	Fe_SA_@N‐G	0	0
4	Fe_SA_@N‐G	88	99
5	FeCl_3_	67	99
6	N‐G	40	99
7	Fe_NP_@N‐G	60	99
8^b)^	Fe_SA_@N‐G	86	99
9^c)^	Fe_SA_@N‐G	59	99
10^d)^	Fe_SA_@N‐G	88	99
11^e)^	Fe_SA_@N‐G	90	99
12^f)^	Fe_SA_@N‐G	17	99
13^g)^	Fe_SA_@N‐G	76	99
14^h)^	Fe_SA_@N‐G	90	99
15^i)^	Fe_SA_@N‐G	90	99
16^j)^	Fe_SA_@N‐G	65	99
17^k)^	Fe_SA_@N‐G	60	99
18^l)^	Fe_SA_@N‐G	13	99

^a)^ Reaction conditions: aniline (1 mmol), *n*‐octanol (4 mmol), catalyst (5 mg, 0.053 wt.% of Fe), *t*‐BuOK (0.5 mmol), 140 °C, 3 h, MW power 90 W;

^b)^ Fe_SA_@N‐G catalyst (10 mg), time;

^c)^ 2 h and;

^d)^ 4 h), temperature;

^e)^ 160 °C and;

^f)^ 120 °C, *t*‐BuOK;

^g)^ 0.25 mmol;

^h)^ 0.75 mmol;

^i)^ *n*‐octanol (8 mmol), base;

^j)^ KOH;

^k)^ NaOH) and;

^l)^ thermal condition (without MW reactor). Conversion and selectivity are determined based on aniline and product analyzed using gas chromatography‐mass spectrometry (GC‐MS) analysis.

### 2.3 Substrate Scope for (hetero)Aromatic and Aliphatic Amines with (hetero)Aromatic and Aliphatic Alcohols

The developed protocol was used for studying the *N*‐alkylation (3a‐an) of different (hetero)aromatic and aliphatic amines with (hetero)aromatic and aliphatic alcohols with Fe_SA_@N‐G catalyst under optimized conditions (**Table** **2**). The initial reaction involved the conversion (88%) of aniline through alkylation with *n*‐octanol, yielding *N*‐octylaniline (3a). The presence of a methyl group in 4‐methyl aniline enhanced the conversion (upto 99%) to 4‐methyl‐*N*‐octylaniline product (3b). Furthermore, the presence of steric hindrance from the o‐methyl group of 2‐methyl aniline did not affect the conversion (99%) to the desired 2‐methyl‐*N*‐octylaniline product (3c). The presence of p‐ and o‐methyl groups enhanced the conversion due to their electron donating effect. Simultaneously, the substitution of an electron‐withdrawing group (4‐methoxy, 2‐aminopyridine, 4‐fluoro, and 3‐chloro) on aniline led to the formation of *N*‐alkylated products (3d‐g) with exceptional conversion and selectivity. Furthermore, the scope of the methodology was explored for *N*‐alkylation of aniline with different aliphatic alcohols. Moreover, the exploration of substituted aniline with *n*‐hexanol resulted in outstanding conversion and selectivity. The desired conversion of 97% to *N*‐hexylaniline (3 h) was achieved through the *N*‐alkylation of aniline with *n*‐hexanol. In contrast, the conversions of 4‐methylaniline and 2‐methylaniline to the corresponding N‐alkylated products (3i and 3j) were lower, due to their reduced reactivity and increased steric hindrance. Anilines bearing various substituents, including 4‐methoxy, 2‐aminopyridine, 4‐fluoro, and 3‐chloro groups were effectively subjected to *N*‐alkylation, resulting in significantly enhanced conversion and excellent selectivity for the desired products (3k–3n).

**Table 2:** Substrate scope for (hetero)aromatic and aliphatic amine with (hetero)aromatic and aliphatic alcohol on Fe_SA_@N‐G catalyst. (see table footnotes).

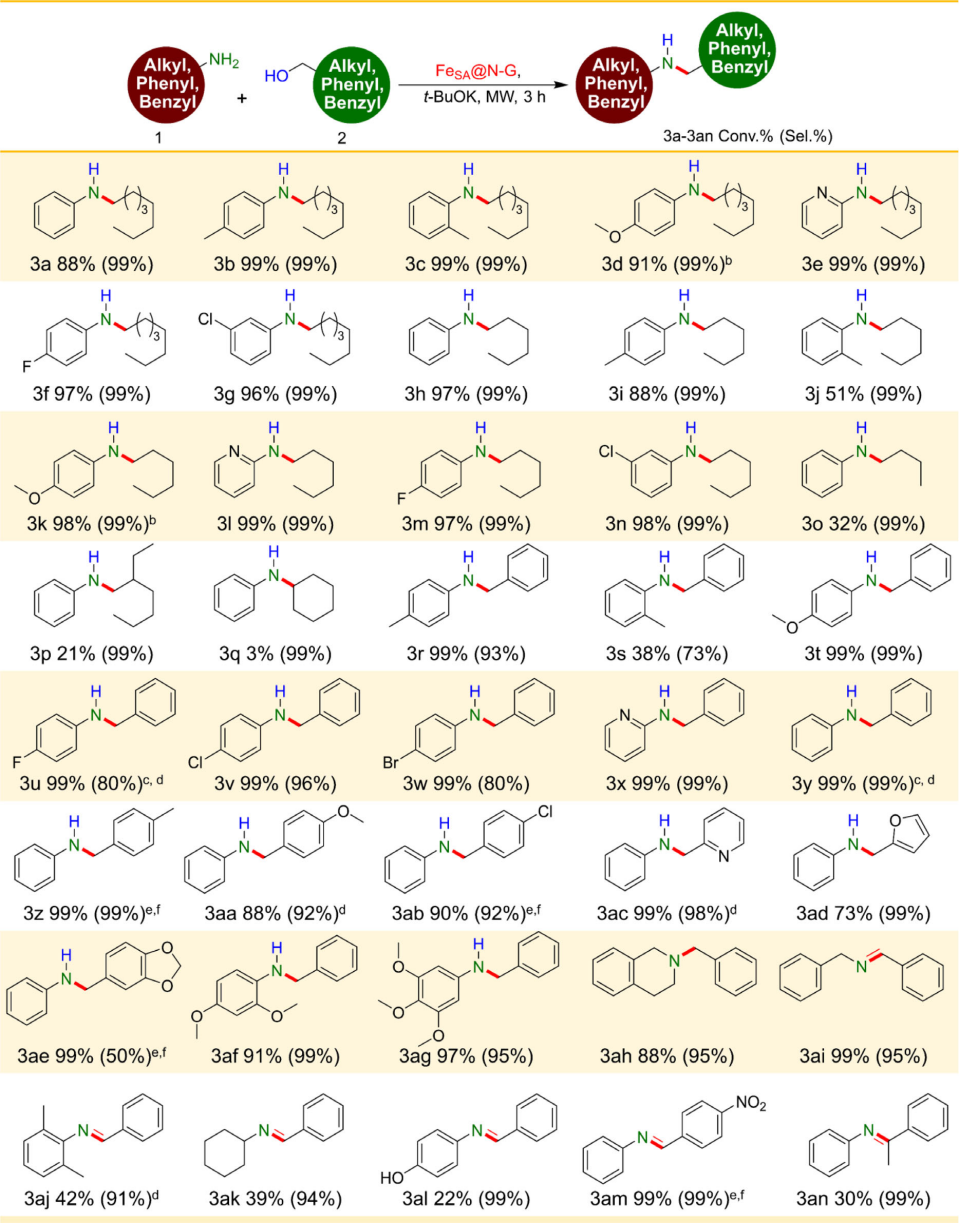

^a)^ Reaction conditions: Amine (1 mmol), aliphatic alcohol (4 mmol), aromatic alcohol (8 mmol), Fe_SA_@N‐G (5 mg, 0.053 wt.% of Fe), *t*‐BuOK (0.5 mmol), MW (temp. 140 °C, power 90 W), 3 h;

^b)^ *n*‐octanol (8 mmol);

^c)^ benzyl alcohol (4 mmol)

^d)^ time (2.5 h);

^e)^ alcohol (1.5 mmol);

^f)^ toluene (1 mL). Conversion and selectivity calculated based on amines and product confirmed by GC‐MS analysis.

Additionally, aniline shows low conversion toward the desired N‐alkylated product (3o‐q); when reacted with different alcohols such as *n*‐butanol, 2‐ethyl hexanol, and cyclohexanol.

We further explored the substrate scope of (hetero)aromatic and aromatic amines with benzyl alcohol. Additionally, the potential for synthesis of aniline derivatives by selective *N*‐alkylation with benzyl alcohol on the Fe_SA_@N‐G catalyst was also evaluated. The aniline compounds with electron‐donating groups (p‐Me and p‐OMe) successfully produced the *N*‐alkylated product with high conversion, i.e., 99% and 99% respectively (3r and 3t). However, when o‐methylaniline was used, the conversion decreased to 38% (3s) due to high steric hindrance caused by the ortho‐Me group. An interesting finding was that the halogenated aniline substrates (‐F, ‐Cl, and ‐Br) displayed effective catalytic conversion for *N*‐alkylation process (3u‐w). However, the substrate with a strong electron withdrawing group (EWG) (p‐nitroaniline) was unsuccessful in obtaining the desired product. The reason behind this phenomenon is the resonance between the lone pair of aniline and the ‐NO_2_ group, which hinders the reaction of aniline with benzaldehyde produced during the reaction.

It is medicinally important to highlight the exceptional performance of the heterocyclic reactant (2‐aminopyridine), which exhibited a 99% conversion with 99% selectivity toward the desired product (3x). Further, we examined the Fe_SA_@N‐G catalyst's broad applicability in selective *N*‐alkylation of aniline with a broad range of (hetero)aromatic and aromatic benzyl alcohol substrates, resulting into *N*‐monoalkylated amine derivatives (Table 2). Initially, we investigated the coupling of aniline with benzyl alcohol and the impact of electron donating functional groups on benzyl alcohol, such as p‐Me and p‐OMe. Notably, the results indicated a remarkable conversion of ≈99%, 99%, and 88% into the formation of N‐alkylated products (3y‐aa). Furthermore, the presence of EWG (p‐Cl) substituted benzyl alcohol leads to a significant increase in conversion and selectivity of the product (3ab).

Interestingly, medicinally important heteroatom‐containing alcohols, such as pyridin‐3‐ylmethanol and furan‐2‐ylmethanol, were able to produce the desired secondary amines (3ac and 3ad) with a high conversion, i.e., 99% and 73% respectively. Additionally, the combination of piperonyl alcohol and aniline resulted into a significant increase in conversion (99%) and a decrease in selectivity (50%) toward the desired secondary amine product (3ae). Additionally, other substituted aniline derivatives were examined, such as 2,4‐dimethoxyaniline and 3,4,5‐trimethoxyaniline, which exhibited outstanding conversion (91% and 97%) with high selectivity toward the targeted products (3af and 3ag). Moreover, the *N*‐alkylation of 1,2,3,4‐tetrahydroisoquinoline displayed exceptional conversion (88%) and selectivity (95%) toward the desired product (3ah). Nevertheless, the conversion of benzylamine, 2,6‐dimethylaniline, cyclohexylamine, and 4‐hydroxyaniline to intermediate imine (3ai‐al) and further did not result in the successful acquisition of the targeted N‐alkylated products. The hydrogen transfer process was hindered due to the weak interaction between Fe single atom sites and imine in cases involving aliphatic amines, hindered aromatic amines, and EWG.^[^ ^18^ ^]^ Furthermore, aliphatic amines exhibit poor nucleophilic reactivity, resulting in a sluggish rate of amine condensation to imines. Nevertheless, the *N*‐alkylation of aniline utilizing p‐nitrobenzyl alcohol, known for its strong electron‐withdrawing ‐NO_2_ group, exhibits exceptional conversion (99%) and selectivity (99%) toward the formation of the intermediate imine (3 am). However, the reaction did not progress to the final product due to resonance occurring between the p‐nitro group and the imine double bond of the intermediate imine.

In the *N*‐alkylation of aniline with 2‐phenylethanol, a secondary alcohol, an imine intermediate (3an) was formed under similar reaction conditions. However, the hydrogenation of this imine intermediate to the *N*‐alkylated product was hindered due to the presence of the methyl group in the secondary alcohol, which impedes the reduction process.^[^ ^22^ ^]^ Although it is worth mentioning that the produced imines (3 am and 3an) serve as valuable intermediates in the synthesis of pharmaceutical drugs and value‐added compounds.^[^ ^59^ ^]^

### 2.4 Synthesis of Pharmaceutically Bioactive Drugs

**Figure 4:**
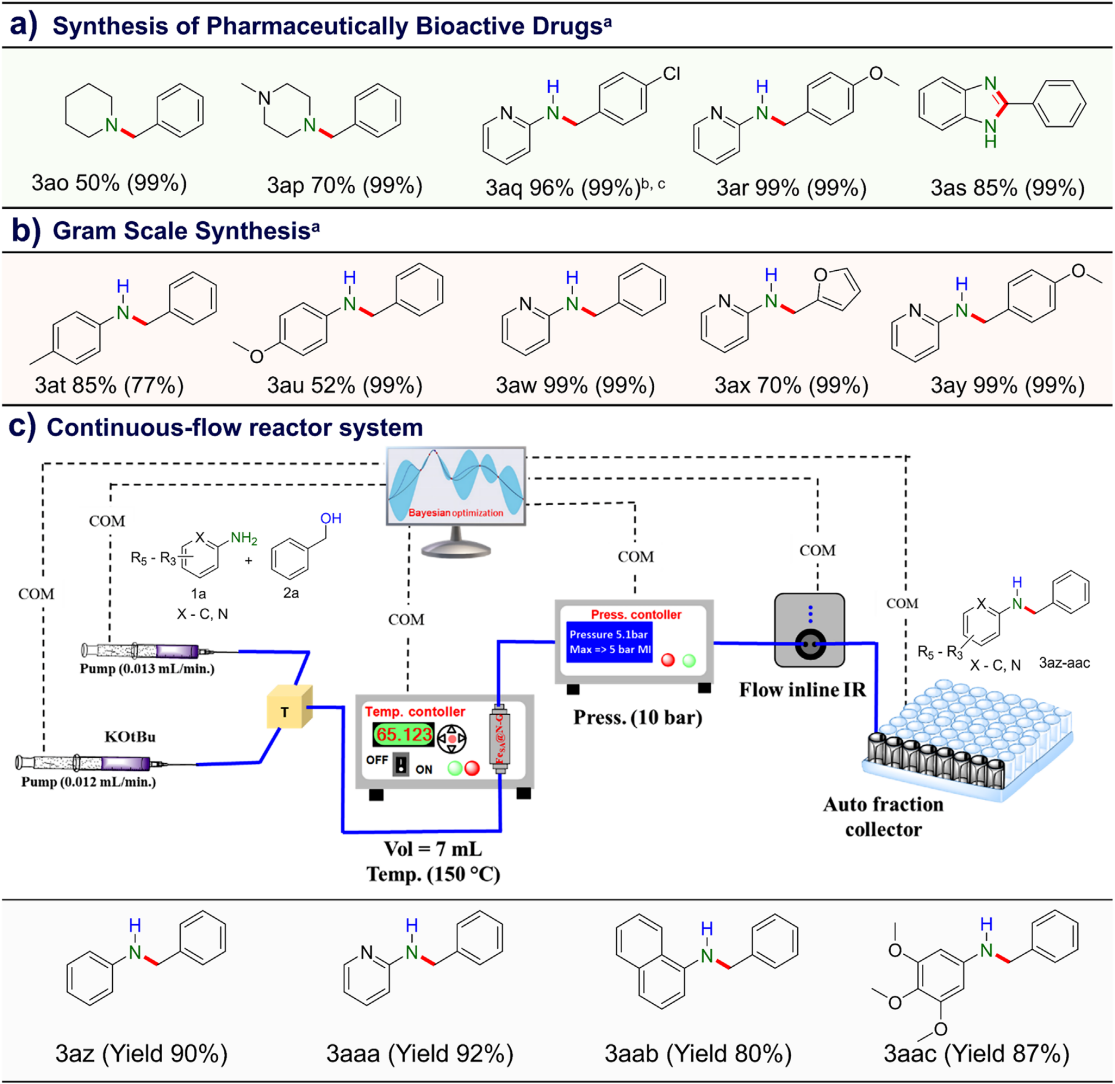
a) Synthesis of bioactive pharmaceutical drugs on Fe_SA_@N‐G catalyst. Reaction conditions: Amine (1 mmol), alcohol (4 mmol), Fe_SA_@N‐G (5 mg, 0.053 wt.% of Fe), *t‐*BuOK (0.5 mmol), MW temp. (140 °C), power (90 W), time (3 h). 4‐chlorobenzyl alcohol (^b^1.5 mmol), ^c^Toluene (1 mL). Conversion and selectivity calculated based on aniline and product confirmed by GC‐MS analysis. b) Fe_SA_@N‐G catalyst enables gram‐scale *N*‐alkylation of aromatic amine with alcohol. Reaction conditions: Amine (10 mmol), alcohol (40 mmol), Fe_SA_@N‐G (50 mg), *t‐*BuOK (5 mmol), MW temp. (140 °C), power (90 W), time (6 h). Conversion and selectivity calculated based on amine and product confirmed by GC‐MS analysis. c) Schematic continuous‐flow reactor system: Scope of substrates for the thermally activated *N*‐alkylation reactions under flow conditions. 0.1 M of compound 1a in benzyl alcohol (2a) + toluene (4:1); 0.1 M solution of *t‐*BuOK in toluene, 7 mL cartridge filled with 1 g of catalyst (Fe_SA_@N‐G), leaving 1.5 mL free space.

We have effectively applied Fe_SA_@N‐G catalyst for the synthesis of pharmaceutically active intermediates and biologically relevant amines including monoamine oxidase inhibitor (3ao)_,_ stimulant drug (3ap), antihistamine (3aq and 3ar) and pharmaceutical intermediate (3as) in good conversion/selectivity with excellent step‐ and atom‐economy under MW conditions using heterogeneous Fe_SA_@N‐G catalyst as shown in **Figure** **4**a.^[^ ^3^, ^60^ ^]^ Typically, these drug molecules and their related compounds are synthesized through reaction sequences that involve reductive amination reactions utilizing specialized catalysts in the presence of H_2_ gas or NaBH_4_ as reducing agents. A notable advantage of the Fe‐catalyzed BH methodology presented in this study is its independence on external reducing agents and suppressed production of halide waste.^[^ ^61^, ^62^ ^]^

### 2.5 Gram Scale Synthesis

The industrial applicability of this methodology was further demonstrated through gram‐scale synthesis of various *N*‐alkylated amines, resulting in moderate conversion and selectivity toward the desired products (Figure 4b, 3at‐ay).

### 2.6 Continuous‐Flow Coupling of Amines with Alcohols on Fe_SA_@N‐G Catalyst

The Fe_SA_@N‐G catalyst was further validated using a catalyzed gram‐scale reaction involving the coupling of an amine with an alcohol. A 10 mmol batch yielded the product with a moderate to high degree of conversion and selectivity for the N‐alkylated product, as demonstrated in Figure 4b (3at‐ay). Subsequently, we expanded the applicability of our methodology by successfully transferring the batch reaction to a continuous flow process. Initially, we optimized various reaction parameters including flow rate, temperature, pressure, and residence time, as summarized in Table S6, entries 1 to 6 in Supporting Information. Following the optimization of the coupling reaction between aniline and benzyl alcohol, an excellent amine conversion of 99% and a yield 90% for the *N*‐benzylaniline product were achieved under the optimal conditions as shown in Table S6, entry 6 in Supporting Information. In addition to the previous findings, we expanded the applicability of the same reaction conditions (Table S6, entry 6, Supporting Information) to a continuous flow system for a comprehensive study of the substrate scope, and the results were exceptionally positive, with remarkably high yields of the *N*‐alkylated products (Figure 4c, 3az‐3aac).

### 2.7 Density Functional Theory (DFT) Study

An 8 × 8 × 1 supercell of graphene with three different types of N_4_ units, shown in Figure S8a–c (Supporting Information), was used as a basic model to describe the Fe‐support coordination site that is consistent with the experimental results. Optimized structures of the Fe_1_‐N_4_ models are shown in Figure S8 (Supporting Information), including four pyridinic‐N (Figure S8a, Supporting Information), four pyrrolic‐N (Figure S8b, Supporting Information), and 2 pyridinic‐N and 2 pyrrolic‐N (Figure S8c, Supporting Information). The measured Fe‐N bond distances in the model, considering all pyridinic nitrogens, are found to be 1.894 Å, which are shorter than the experimentally observed value in Figure S8d (Supporting Information). In the model considering Fe bound to all pyrrolic‐N, the corresponding Fe‐N distances are measured to be 2.280 and 2.338 Å, which are larger than the experimental values (Figure S8e, Supporting Information).

**Figure 5:**
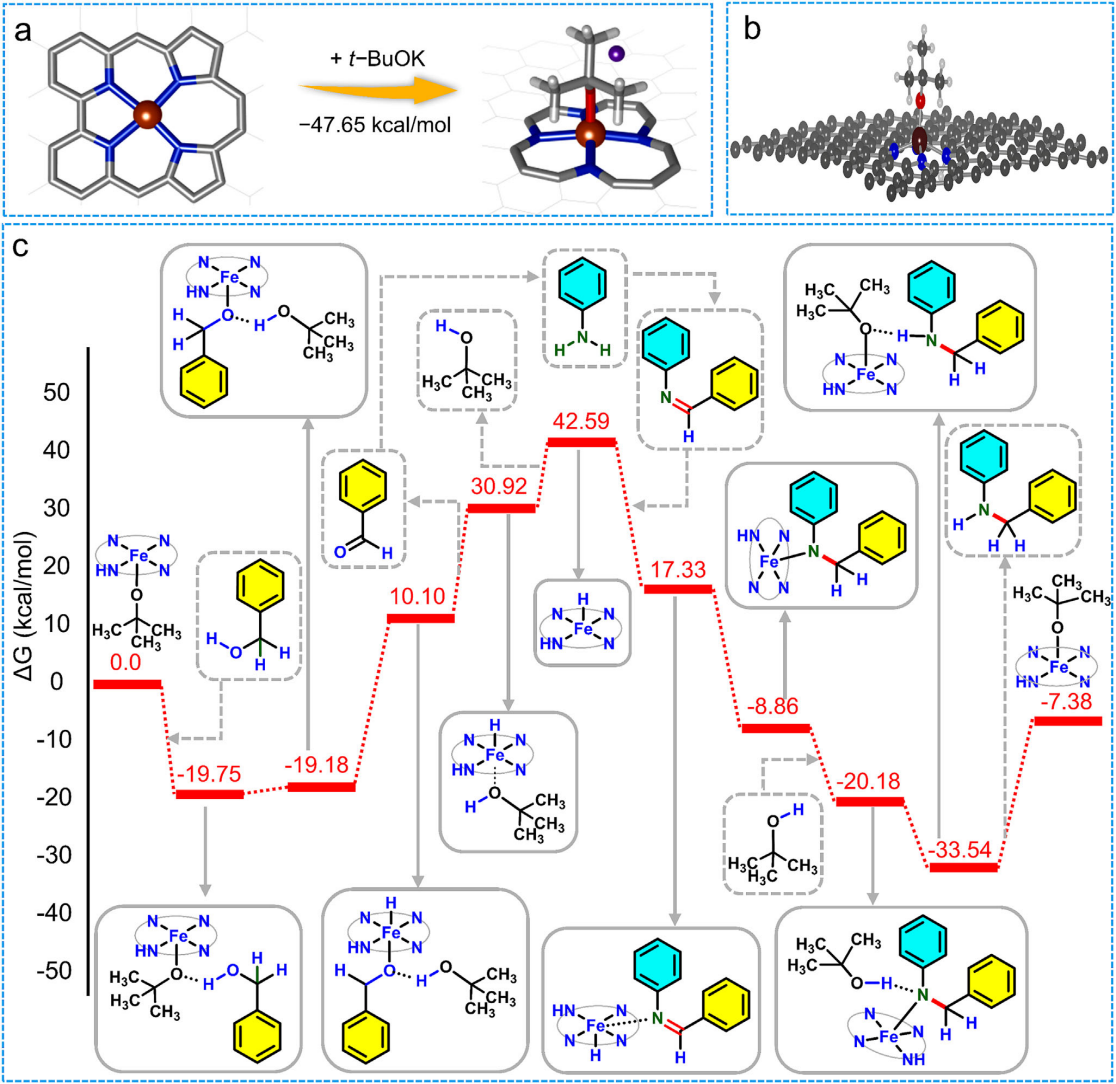
a) Adsorption of *t‐*BuOK over the Fe‐N_4_ active site of the catalyst substrate. b) Model of *t*‐BuO bound FeN_4_ embedded in graphene. c) DFT study: dehydrogenation of alcohol followed by C = N coupling and hydrogenation of imine to achieve N‐alkylated amine.

In the model shown in Figure S8f (Supporting Information), Fe is found to be four N‐coordinated (2 pyridinic‐N and 2 pyrrolic‐N) with Fe‐N bond distances of 1.988 and 2.117 Å, which are closer to the experimental results, and hence this system is considered for all further studies. Calculated distance between the two pyrrolic‐N sites was found to be 2.875 Å, while that between pyridinic‐N and two pyrrolic‐N was 2.67 Å. The calculated Fe metal atom binding energy at the N_4_ cavity is found to be ‐68.08 kcal mol^−1^, indicating the stability of the bound metal against the metal aggregation and forming clusters/NPs. In the considered Fe_1_‐N_4_ system, Fe is found to be in a quartet spin state with three unpaired electrons, confirming its 3+ oxidation state. We considered the *t‐*BuOK adsorption over the Fe‐N_4_ site as reported in **Figure** **5**a, and the optimized structure is found to have a Fe−O distance of 1.929 Å, and the adsorption energy is calculated to be ‐47.65 kcal mol^−1^.

Based on experimental and DFT studies, the recently reported mechanistic findings regarding the Fe‐catalyzed *N*‐alkylation reaction of amines with alcohol,^[^ ^63^ ^]^ we illustrated the plausible catalytic pathway (Figure 5c). To calculate the reaction energy profile for the proposed mechanism from DFT studies, *t*‐BuO‐bound FeN_4_ embedded graphene is considered as the catalyst, and to make the system charge neutral, one of the pyrrolic N is hydrogenated as shown in Figure 5b. In order to calculate the reaction energies for the intermediate reactions depicted in Figure 5c, the minimum energy configurations of the respective reaction intermediates were optimized. The corresponding energy profile can be observed in Figure 5c. In the proposed reaction mechanism, first, Fe_SA_@N‐G in the presence of base (promoter) dehydrogenates the alcohol to aldehyde, and subsequently, Fe metal borrows hydrogen atom (Fe‐H) (Figure 5c). Afterward, the aldehyde and amine undergo a condensation reaction, resulting in the formation of the imine intermediate. Lastly, the transferred hydrogen of the imine intermediate utilizing Fe‐H_1_ formed in the first step, selectively generates the corresponding *N*‐alkylated amine product (Figure 5c).

### 2.8 Recyclability Study

**Figure 6:**
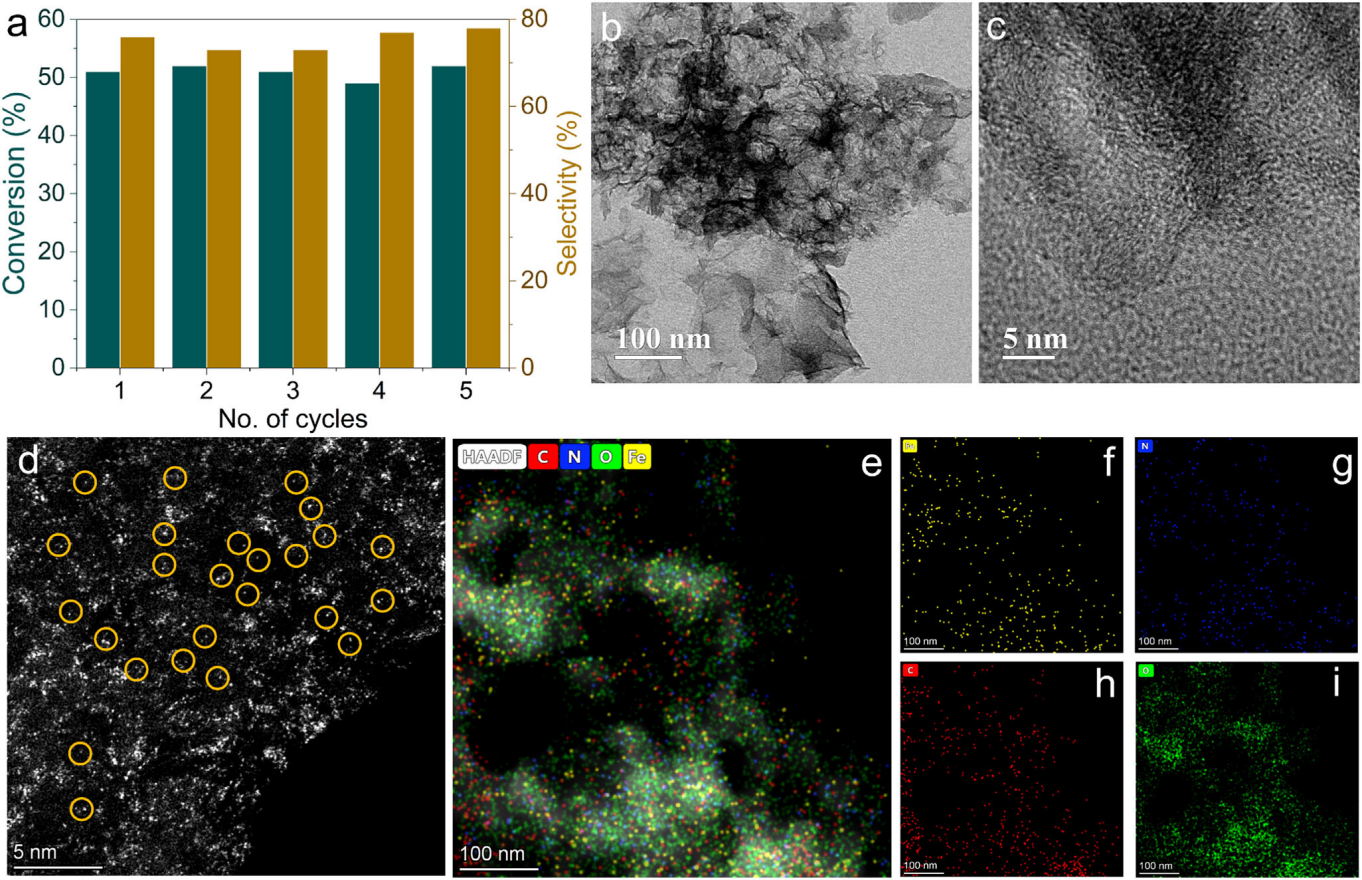
a) Reusability study of Fe_SA_@N‐G catalysts for coupling of aniline with benzyl alcohol at a gram scale. b‐c) HR‐TEM images of reused Fe_SA_@N‐G. d‐i) HAADF‐STEM image and elemental mapping of Fe_SA_@N‐G.

An examination of the recyclability of the Fe_SA_@N‐G catalyst was performed on a gram scale for the *N*‐alkylation of aniline with benzyl alcohol, resulting in conversion (52%) and selectivity (78%). After the reaction was completed (6 h), the Fe_SA_@N‐G catalyst was isolated through centrifugation, followed by three washes with ethyl acetate. The catalyst was then dried in a high vacuum oven at 80 °C for 12 h and subsequently reused for the next reaction cycle. **Figure** **6**a demonstrates that there were no noteworthy changes in catalytic conversion and selectivity toward the *N*‐benzyl aniline product, even after five cycles.

The XRD pattern of both fresh and reused Fe_SA_@N‐G catalyst (after 5 cycles) revealed no discernible changes in the two broad peaks depicted (Figure S9a, Supporting Information). After that, we analyzed the recovered Fe_SA_@N‐G catalyst using XPS. The elemental composition (Fe, N, C, and O), valence states of iron as well as the bonding characteristics of N, C, and O were fully preserved (Figure S9b–f, Supporting Information). Furthermore, after five catalytic cycles, the morphology of the catalyst was just slightly changed, but importantly, no aggregates or Fe‐based clusters were observed in the reused Fe_SA_@N‐G catalyst (Figure 6b,c). Finally, the reused Fe_SA_@N‐G catalyst was thoroughly examined using HAADF‐STEM, revealing a uniform distribution of Fe single‐atoms on the N‐G support (Figure 6d–i).

## 3 Conclusion

In conclusion, we have developed a simple ion adsorption strategy to produce an N‐doped graphene‐supported Fe single‐atom catalyst (Fe_SA_@N‐G) that is highly stable, active, and selective for the *N*‐alkylation of amines with alcohols. This innovative catalyst demonstrated exceptional catalytic performance, achieving record TON and TOF values in *N*‐alkylation reaction under solvent‐free conditions, surpassing all previously reported protocols (Table S1, Supporting Information). We successfully tested over 50 substrates, showcasing the catalyst's versatility in synthesizing a wide range of N‐alkylated products, including stimulant drugs, antihistamines, and other biologically active compounds. We also explored the reaction mechanism covering several steps, namely i) the *t*‐BuO bound with Fe_1_‐N_4_ complex is effective for dehydrogenation of alcohol, ii) Fe_1_^(III)^−N_4_ serves as the active site for the borrowing hydrogen, and iii) the Fe_1_−H single atom demonstrates effectiveness in transferring hydrogen to imine intermediates. Notably, the catalyst exhibited excellent recyclability, scalability (up to gram‐scale quantities), and performance under continuous flow conditions, highlighting its significant potential for immediate application in industrial processes.

## Conflict of Interest

The authors declare no conflict of interest.

## Supporting information



Supporting Information

## Data Availability

The data that support the findings of this study are available from the corresponding author upon reasonable request.
