# Supplementary material for: Iron Single‐Atom Catalyzed N‐Alkylation of Amines with Alcohols via Solvent‐Free Borrowing Hydrogen Strategy
Source: Adv Sci (Weinh). 2025 Aug 19;12(46):e07915. doi: 10.1002/advs.202507915 (PMC12697845; doi:10.1002/advs.202507915)
Supplement: Supplementary file 1 — Supporting Information [file ADVS-12-e07915-s001.docx]

**Supporting Information**

**Iron Single-Atom Catalyzed N-Alkylation of Amines with Alcohols via Solvent-free Borrowing Hydrogen Strategy**

Arun D. Kute,^[a]^ Hanumant B. Kale,^[a]^ Priti Sharma,^[b]^ Srinivasu Kancharlapalli,^[c]^ Gajanan Y. Shinde,^[a]^ Ruchi Chauhan,^[d]^ Ajay K. Singh,^[d]^ Shan Jiang,^[e]^ Jeffrey T. Miller,^[e]^ Radek Zboril,^[b],[f]*^ Yifeng Wang,^[g]*^ and Manoj B. Gawande^[a], [f]*^

^[a]^Department of Industrial and Engineering Chemistry, Institute of Chemical Technology, Mumbai- Marathwada Campus, Jalna-431213 Maharashtra, India.

^[b]^Regional Centre of Advanced Technologies and Materials, Czech Advanced Technology and Research Institute, Palacký University, Olomouc 27, Šlechtitelů, 779 00, Czech Republic.

^[c]^Chemistry Division, Bhabha Atomic Research Centre, Mumbai 400085, India.

^[d]^Department of Organic Synthesis and Process Chemistry, CSIR-Indian Institute of Chemical Technology, Hyderabad, 500007, India.

^[e]^ Davidson School of Chemical Engineering, Purdue University, 480 Stadium Mall Drive, West Lafayette, IN 47906, United States.

^[f]^Nanotechnology Centre, Centre for Energy and Environmental Technologies, VŠB–Technical University of Ostrava, 17. Listopadu 2172/15, Ostrava-Poruba, 708 00, Czech Republic.

^[g]^Department of Chemistry and Chemical Engineering, Shandong University, Shandong Province, PR China.

*Corresponding authors: Radek Zbořil (radek.zboril@upol.cz), Yifeng Wang (yifeng@sdu.edu.cn) and Manoj B. Gawande (mb.gawande@marj.ictmumbai.edu.in).

1. **Materials and Methods**

**1.1 Instrumentation**

Powder X-ray diffraction patterns (XRD) were recorded using a Bruker D8 Advance X-ray diffractometer equipped with a Cu-Kα radiation source (λ = 1.5418 Å). Raman analysis was performed with an InVia Raman microscope (Renishaw). Infrared spectroscopy was performed using a FT/IR 6600 JASCO. The imaging of catalyst morphology was obtained through transmission electron microscope (TEM), high resolution TEM (HR-TEM), high-angle annular dark-field scanning transmission electron microscope (HAADF-STEM) and energy dispersive X-ray spectroscopy (EDS) elemental mapping operating at 200 kV. Chemical compositions and states were analyzed using X-ray photoelectron spectroscopy (XPS, Thermo Scientific, ESCALAB 250 XI+). The Fe K edge X-ray absorption spectroscopy (XAS) experiment was conducted at 10 BM of the advanced photon source, Argonne National Laboratory (ANL). BET surface area and pore size measurements were performed using N_2_ adsorption/desorption isotherms at 77 K on a BELSORP MAX II instrument. Gas chromatography mass spectroscopy (GC-MS) was utilized to identify desired products. The progress of the reaction was monitored at different time intervals and quantified by GC-MS (SHIUMADZU GC-MS-QP2020) equipped with an SH-Rxi-5Sil MS column (0.25 mm × 0.25 um × 30 m). Analytical thin-layer chromatography (TLC) was performed using analytical chromatography silica gel 60 F254 precoated plates (0.25 mm). The developed chromatogram was analysed by UV lamp (365 nm). PTFE tubing (id = 1000 μm), T-junction, and high-purity PFA tubing were purchased from Upchurch IDEX HEALTH & SCIENCE. A homemade batch reactor, purchased from Lelesil Mumbai, India, was slightly modified for the continuous flow reaction.

**1.2 Materials and chemicals:**

Most of the reagents and chemicals were purchased from Sigma-Aldrich and were used as such without any further purification. Common organic chemicals and salts were purchased from Avra chemicals, India. De-ionized (DI) water was used in all experiments wherever required. All work-up and purification procedures were carried out with reagent-grade solvents.

1. **Experimental Section**

**2.1 Synthesis of N-doped graphene (N-G)**

The procedure involved drying 20 g of dicyandiamide in an oven at a temperature of 90 ^o^C for a duration of 12 h. Subsequently, 20 g of the oven-dried powder of dicyandiamide was placed in a boat crucible and heated to 550 °C at a rate of 5 °C min^-1^, then maintained at that temperature for 2 h. The resulting yellow material was referred as g-C_3_N_4_. In the next step, 10 g of g-C_3_N_4_ was dispersed ultrasonically for 30 min in a 20 gm glucose aqueous solution (200 ml), followed by freeze-drying for 30 h. The resulting freeze-dried powder (10 g), was then subjected to tube furnace at 950 °C in an N_2_ atmosphere for 1 h, with a heating rate of 5 °C min^-1^ to produce the final product, N-doped graphene (Yield=1.8 g).

**2.2 Synthesis of Fe single-atom over N-doped graphene (Fe_SA_@N-G)**

To synthesize Fe single atoms over N-doped graphene; 500 mg N-doped graphene was dispersed in 200 mL deionized water under high-speed sonication for 4 h Iron (III) nitrate nonahydrate (Fe(NO_3_)_3_·9H_2_O) in DI water (50 mg, 20 mL) was added dropwise to the dispersed N-doped graphene, followed by sonication for 30 min. Subsequently, the mixture was stirred for 12 h, after the addition of Fe(NO_3_)_3_·9H_2_O. The suspension was then stirred for an additional 12 h at 90 °C. The solution was cooled to room temperature (RT) and a fresh aqueous solution of sodium borohydride (NaBH_4_) (2.0 g in 10 mL of DI water) was added dropwise. The reaction mixture underwent stirring for another 12 h at 80 °C, followed by 20 runs (each of 1 min) of rapid microwave heating (LG, power 900 W). The final product was washed twice with DI water using high-speed centrifugation and placed in a freeze dryer for 24 h to yield the final product (Yield = 465 mg).

**2.3 Synthesis of Fe nanoparticle over N-doped graphene (Fe_NP_@N-G)**

500 mg N-doped graphene was dispersed in 200 mL DI water under high-speed sonication for 4 h. Fe(NO_3_)_3_·9H_2_O in DI water (50 mg, 20 mL) was added dropwise to the N-doped graphene (500 mg), and the mixture was sonicated for 30 min. Subsequently, the solution was stirred for 12 h upon the addition of Fe(NO_3_)_3_·9H_2_O. Afterward, the suspension was stirred for an additional 2 h at 90 °C. At room temperature, a fresh aqueous solution of sodium borohydride (NaBH_4_) (2.0 g in 10 mL of DI water) was added dropwise, and the reaction mixture was stirred for another 12 h at 80 °C. The final product was washed with DI water and methanol twice using high-speed centrifugation. Subsequently, the product was placed in a high vacuum oven for 24 h before obtaining the final product. (Yield = 450 mg).

**2.4 General procedure for the N-alkylation of amines with alcohols**

In a typical reaction procedure, amine (1 mmol), alcohol (4 mmol), t-BuOK (0.5 mmol) and Fe_SA_@N-G (5 mg) were placed in a microwave vial (35 mL). The reaction mixture (RM) was then heated in the microwave at 140 ℃ (90 W) for 3 h. After completion of the reaction, the microwave vial was allowed to cool down to RT. The RM was centrifuged, and the solution was separated and diluted with 1 mL DI water and then 1 mL ethyl acetate was added to the solution. The extracted ethyl acetate was dried with sodium sulphate. Subsequently, 50 μL of the dried RM was diluted with fresh ethyl acetate and analyzed using GC-MS. (The dynamic mode was used in the microwave synthesizer CEM Discover SP model).

**2.5 A gram scale procedure for N-alkylation amines with alcohols**

In a typical reaction procedure, amine (10 mmol), alcohol (40 mmol), *t*-BuOK (5 mmol) and Fe_SA_@N-G (50 mg) were placed in a microwave vial (35 mL). The RM was heated in the microwave for 6 h at 140 ℃ (90 W), as shown in **Figure S1**. After the reaction was complete, the microwave vial was cooled down to RT. The RM was then centrifuged and the separated solution was diluted with DI water (10 mL) and followed by addition of ethyl acetate (10 mL). The extracted ethyl acetate was dried with sodium sulphate. A 50 μL aliquot of the dried RM was diluted with fresh ethyl acetate and analyzed by GC-MS. (The dynamic mode was used in the microwave synthesizer CEM Discover SP model).

**2.6 Continuous flow procedure for the N-alkylation of amines with alcohols**

To prepare the stock solution of the amine compound (1a**),** 0.455 mL)4.9 mmol) was dissolved in 50 mL of a mixture of alcohol (2a) and toluene (4:1), which was charged into one syringe. Another syringe was loaded with 0.56 g, (5.0 mmol) of the base potassium tert-butoxide dissolved in 50 mL of toluene. These two syringes were connected via pump, with output further connected to a T-mixer. The flow rate of 1a + 2a was set at 0.013 mL/min while the flow rate of the base was set 0.012 mL/min to maintain a stoichiometry of 1:1. The mixture was then passed through a stainless steel cartridge (id = 7 mm, l = 150 mm, vol. = 7 mL) filled with 1 g of catalyst (Fe_SA_@N-G), leaving 1.5 mL of free space, at 150 °C, under 10 bar pressure. The first 2 h out coming of the product mixture (3az-3aac) was discarded and next 12 h of the product mixture [18 mL;1a (83 μL)] was collected in a conical. The reaction mixture was then extracted and separated through a regular batch process. The organic phase was concentrated under reduced pressure to obtain the product.


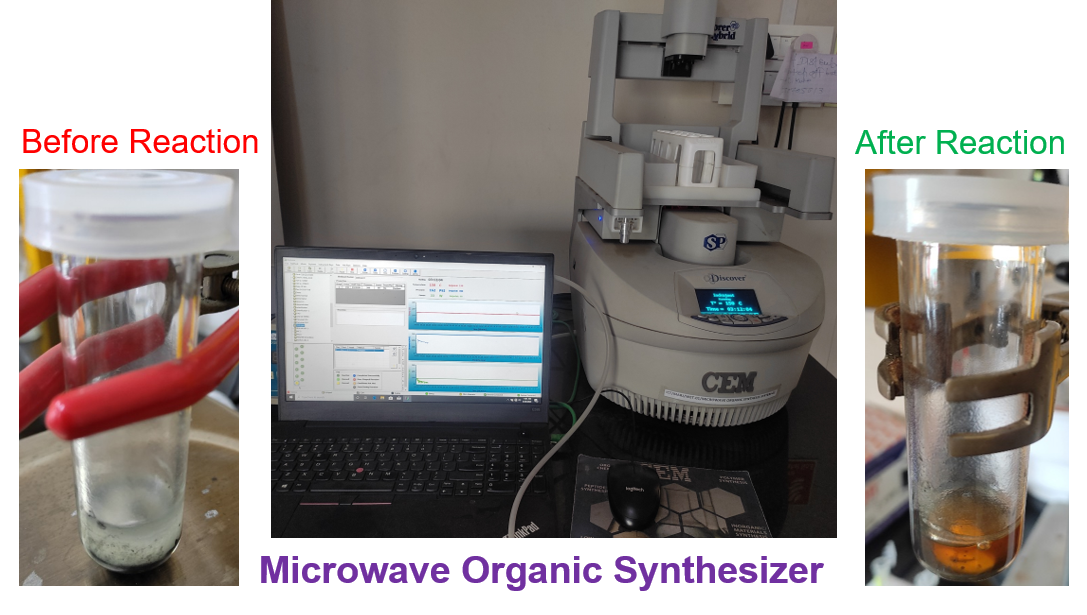


**Figure S1.** Microwave synthesizer used for N-alkylation of amine with alcohol on Fe_SA_@N-G catalyst.

**2.7 Recyclability procedure for N-alkylation of aniline with benzyl alcohol**

In a typical reaction procedure, aniline (10 mmol), benzyl alcohol (40 mmol), *t*-BuOK (5 mmol) and Fe_SA_@N-G (50 mg) were placed in a microwave vial (35 mL). The RM was heated under microwave irradiation for 6 h at 140 ℃ (90 W). Following the completion of the reaction, the microwave vial was cooled down to RT. After centrifuging the RM, the catalyst was washed with ethyl acetate (three times), dried in a high vacuum oven at 80 ^o^C for 12 h, and reused for the next reaction cycle. The supernatant solution was diluted with DI water (10 mL) followed by the addition of ethyl acetate (10 mL). The extracted ethyl acetate RM was dried using sodium sulphate. Next, a 50 μL solution was further diluted with fresh ethyl acetate and subjected to analysis using GC-MS. (The dynamic mode was used in the microwave synthesizer CEM Discover SP model).

**2.8 Product analysis by GC-MS**

The area percent method was utilized to calculate the conversion and selectivity of the reactions. The peak areas of the unreacted starting material, desired products, and by-products were obtained through GC-MS analysis.

**
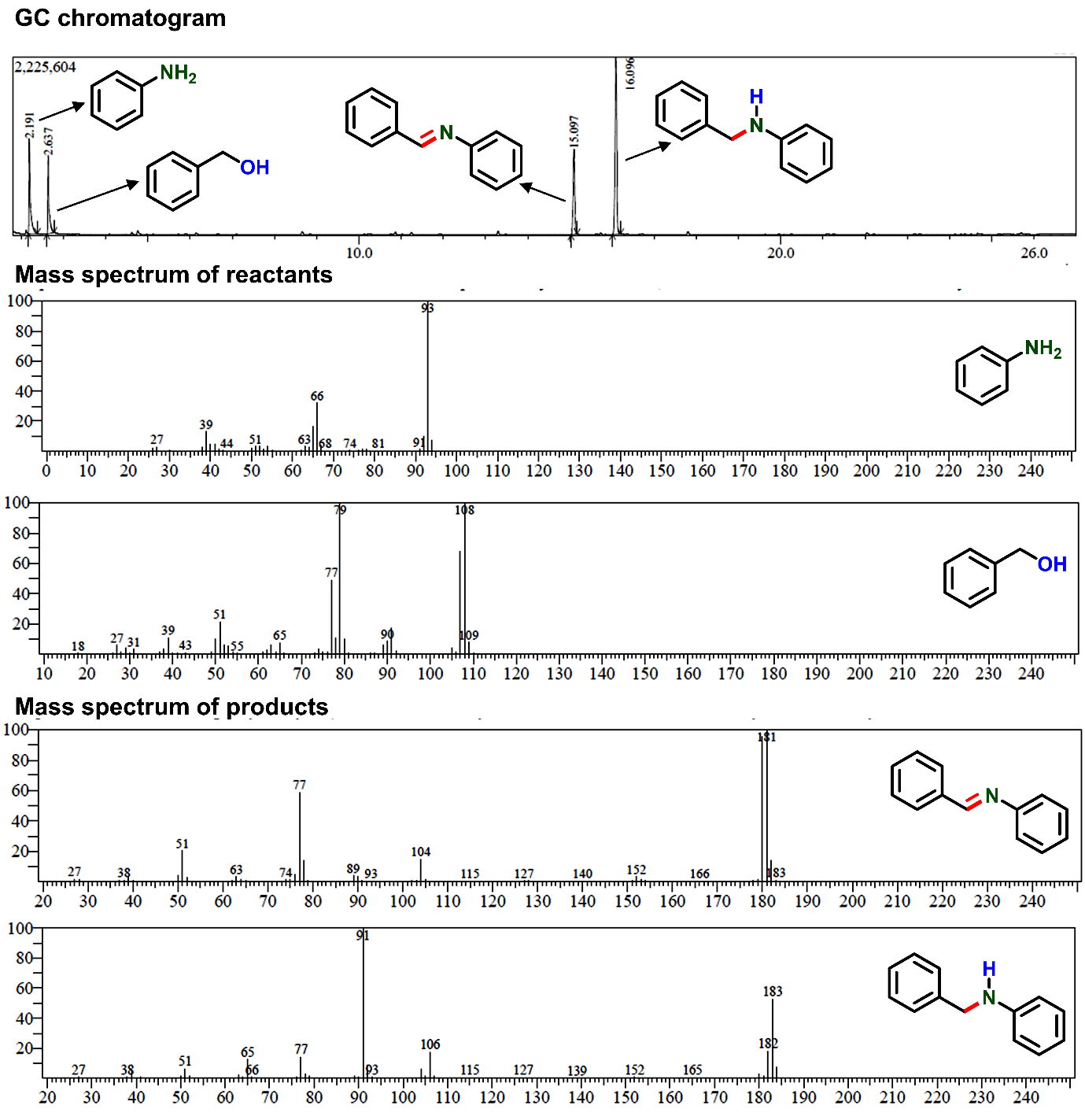
**

**Note:** To validate our results, we repeated the *N*-alkylation reaction using mesitylene as an unreactive internal standard. The comparison of data obtained with and without the internal standard revealed no significant differences in conversion and selectivity, thereby confirming the consistency and reliability of our measurements.

**2.9 Calculations of TON and TOF^[1]^**

**Turnover number (TON) = N_r_ × (C%) × (S%) / 10^4^ × Nc**

**Turnover frequency (TOF) = TON/time (t)**

Nr = Initial moles of substrate, C% = conversion of substrate, S% = Selectivity of desired product and Nc = moles of catalyst or moles of active sites

1. **Characterization of catalysts**


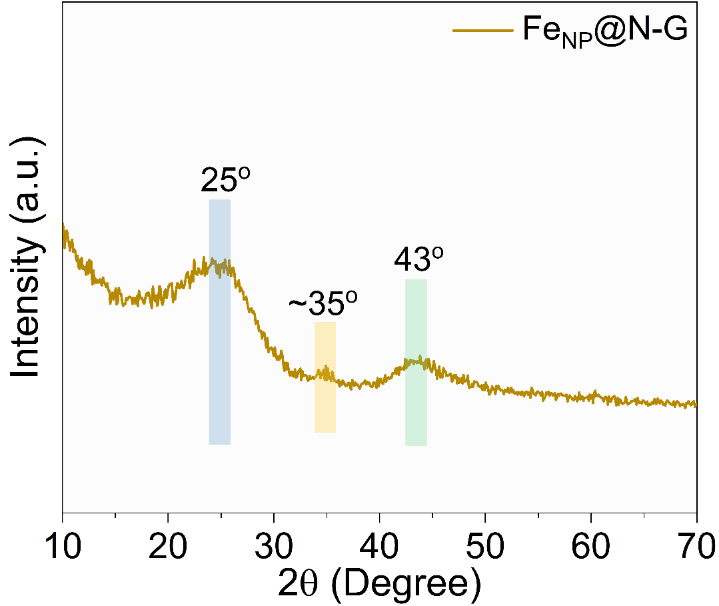


**Figure S2.** XRD pattern of Fe_NP_@N-G.

The XRD patterns of the Fe_NP_@N-G sample revealed the presence of two broad peaks at approximately 25^o^ and 43^o^, attributed to the (002) and (101) planes of N-doped graphene or graphitic carbon materials (**Figure S2**).^[2]^ Additionally, a small peak at around 35° was observed in the Fe_NP_@N-G sample, corresponding to the main (311) diffraction in the maghemite/magnetite structure (**Figure S2**).^[3, 4]^


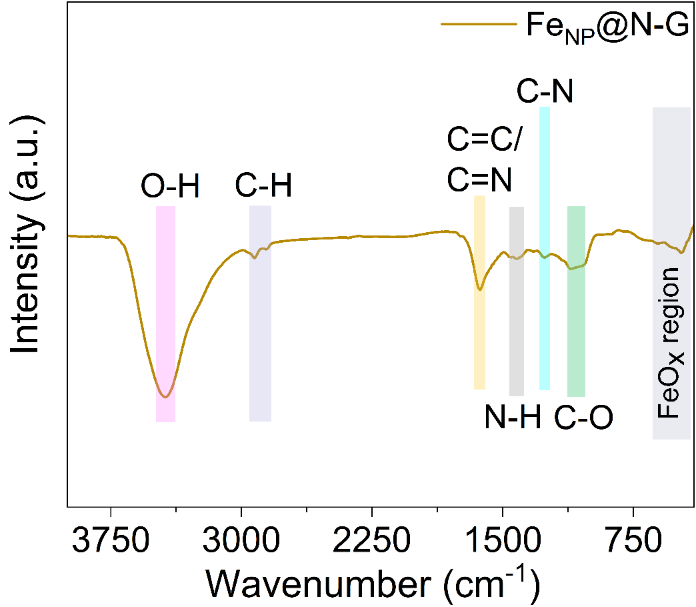


**Figure S3.** FT-IR spectra of Fe_NP_@N-G.

Fourier transform infrared (FT-IR) spectra show distinct stretching vibration peaks at ∼3437 (O-H), ∼2925 (C-H), ∼1628 (C=N/C=C), ∼1418 (N-H), ∼1258 (C-N), and ∼1105 cm^−1^ (C-O), which represent the main chemical groups involved in the structure of the N-G support. Additionally, Fe_NP_@N-G exhibits a low-intensity vibration at approximately 475 cm^−1^, confirming the presence of iron oxide species (**Figure S3**)^[5]^.


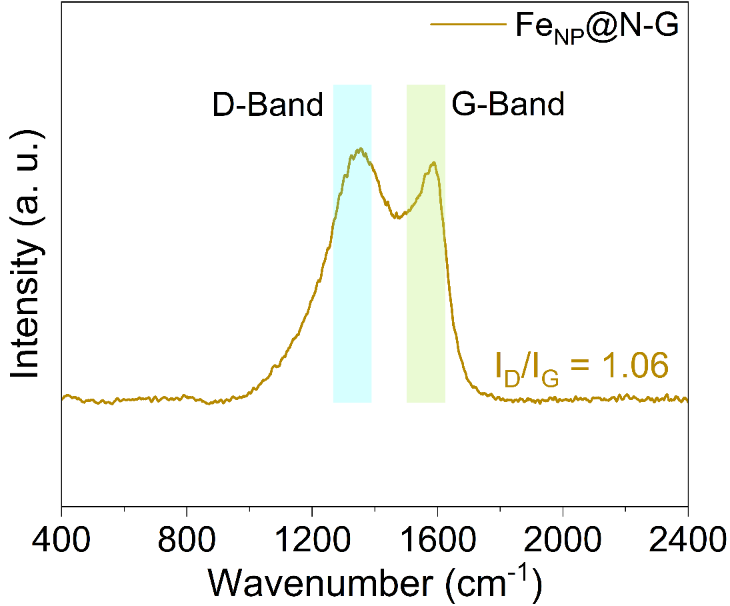


**Figure S4.** Raman spectra of Fe_NP_@N-G.

The Raman spectra of Fe_NP_@N-G display two distinct peaks at around 1329 cm^−1^ and 1586 cm^−1^, corresponding to the D band and G band, respectively. The I_D_/I_G_ ratio of Fe_NP_@N-G is 1.06 (**Figure S4**).^[6, 7]^


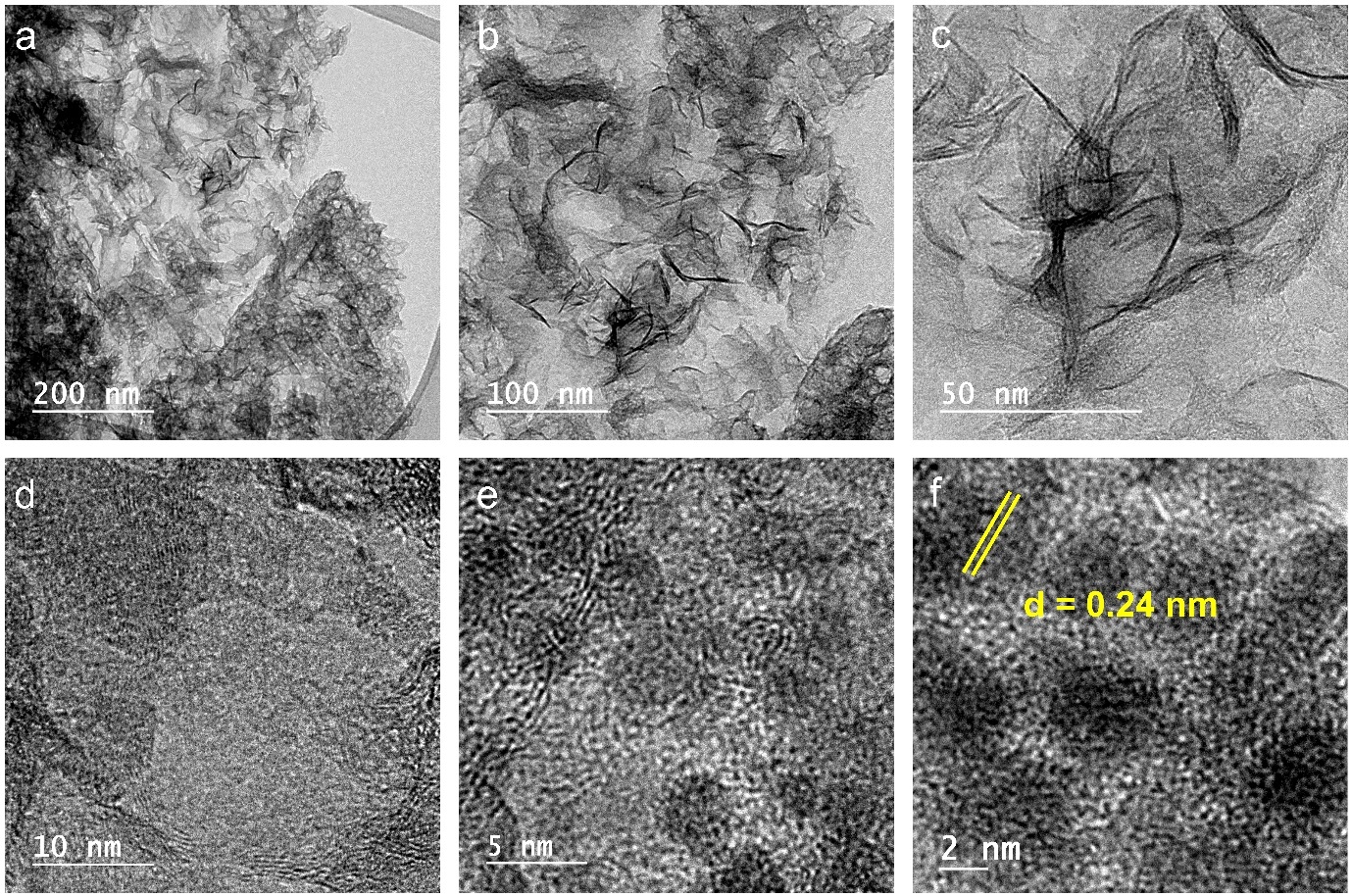


**Figure S5.** a-f) HR-TEM images of Fe_NP_@N-G catalyst.

The morphology of the Fe_NP_@N-G catalyst was analyzed using high-resolution transmission electron microscopy (HR-TEM). Based on the HR-TEM images, it can be deduced that the catalyst exhibits a sheet-like morphology, along with the presence of Fe nanoparticles (NPs) that have an interplanar distance measuring 0.24 nm. (**Figure S5**).


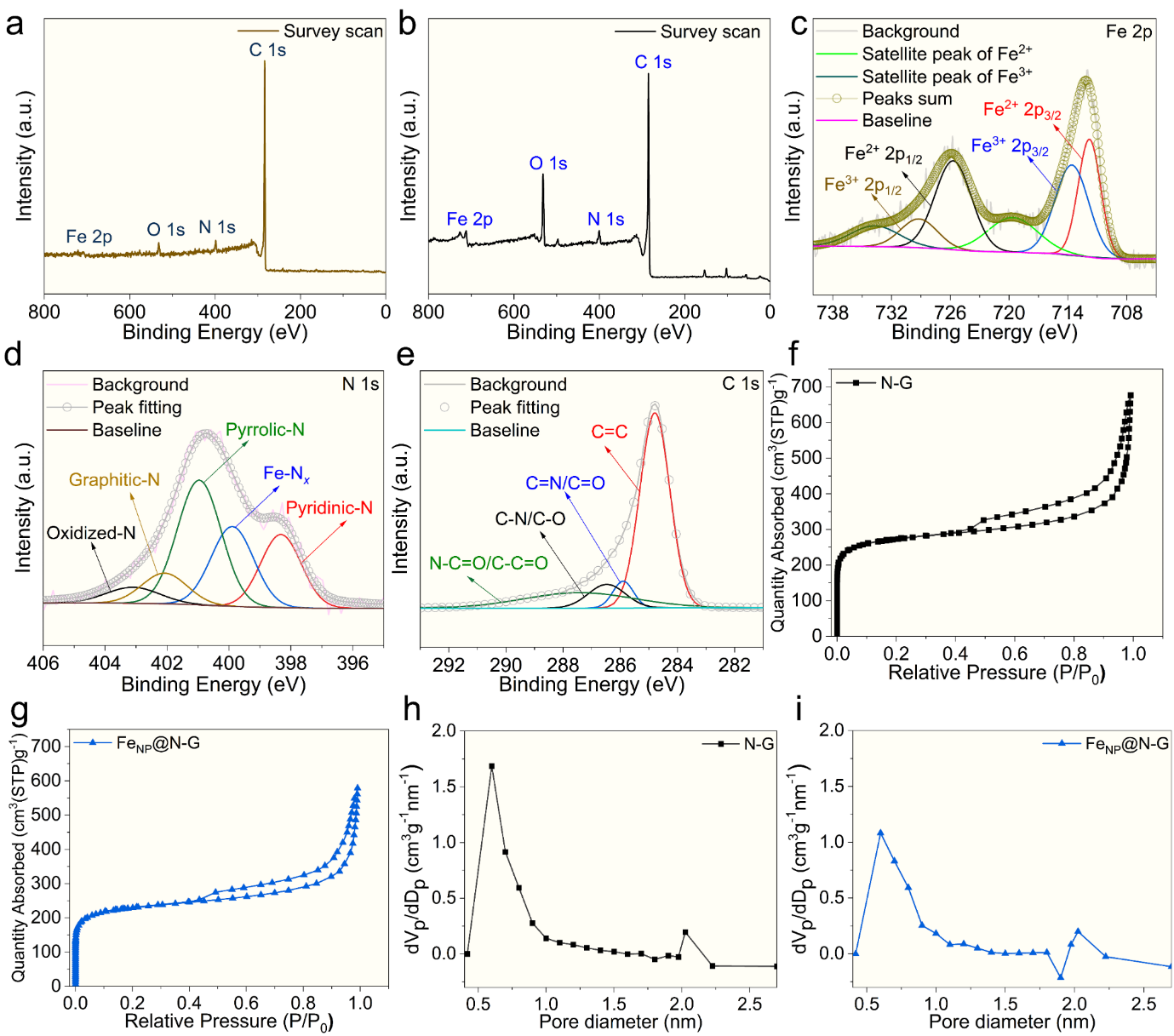


**Figure S6.** Survey scan: a) Fe_SA_@N-G, b) Fe_NP_@N-G. XPS spectra of Fe_NP_@N-G: c) Fe 2p, d) N 1s, e) C 1s. BET surface area and pore size analysis: Nitrogen (N_2_) adsorption–desorption isotherms of f) N-G, g) Fe_NP_@N-G. MP plot: pore diameter of h) N-G, i) Fe_NP_@N-G

The XPS survey scan demonstrated the presence of Fe, C, N, and O elements in Fe_NP_@N-G (**Figure S6b**). The presence of four distinct peaks in (**Figure S6c**) at binding energies of 712.16 eV and 727.03 eV suggests a higher oxidation state of Fe^3+^, in contrast to the peaks at 710.19 eV and 723.63 eV, which indicate the Fe^2+^.^[8] [6, 9] [10]^ The deconvolution of N 1s spectrum revealed that the coexistence of pyridinic-N (397.38 eV), Fe-N_x_ (398.58 eV), pyrrolic-N (400.05 eV), graphitic-N (401.29 eV), and oxidized-nitrogen species (403.06 eV) (**Figure S6d**).^[6, 11]^ The deconvolution of the C 1s spectra revealed four distinct peaks, including, 284.84 eV, 286.79 eV, 288.14 eV, and 289.75 eV which are assigned to C=C (sp^2^), C=O/C=N, C-O/C-N, and N-C=O/C-C=O respectively (**Figure S6e**). The elemental composition and fitting results of Fe_NP_@N-G and Fe_SA_@N-G are presented in **Table S2** and **Table S3**. According to the Brunauer–Emmett–Teller (BET) measurements, the surface areas of the N-G and Fe_NP_@N-G samples were 944 m^2^ g^-1^ and 614 m^2^ g^-1^, respectively (**Figure S6f-g**, **Table S5**). Analysis of the N_2_ isotherms and corresponding pore diameters (0.92 nm, and 0.58 nm) reveals the microporous nature of all the samples (**Figure S6h-i**, **Table S5**).

**Table S1.** Compariosn of Fe_SA_@N-G activity for N-alkylation reaction with previously reported heterogenous catalysts.

| **Sr. No.** | **Starting substrates** | **Catalyst** | **Solvent/ Gas environment** | **Base** | **Temp.**  **(^o^C)** | **Time**  **(h)** | **Conv.**  **(Yield)** | **TON** | **TOF**  **(h^-1^)** | **Ref.** |
| --- | --- | --- | --- | --- | --- | --- | --- | --- | --- | --- |
| **1** | **Aniline (1 mmol) and Benzyl alcohol (4 mmol)** | **Fe_SA_@N-G (5 mg)** | **-** | ***t*-BuOK (0.5 mmol)** | **140** | **2.5** | **99% (99%)** | **1032.7** | **413.1** | **This work** |
| 2 | Aniline (0.2 mmol) and Benzyl alcohol (0.3 mmol) | UiO-66-PPh_2_-Ir  (3 mg) (0.6 mol% of Ir) | Dioxane (2 mL) | *t*-BuOK (0.4 mmol) | 110 | 12 | 95%  (86%) | 2.73 | 0.23 | ^[12]^ |
| 3 | Aniline (0.5 mmol) and Benzyl alcohol (5 mmol) | Zn–N_4_–C (20 mg) | Hexane (20 mL)/ N_2_ (10 bar) | NaNH_2_ (1 equiv.) | 180 | 20 | 92% | 74.2 | 3.7 | ^[13]^ |
| 4 | Aniline (1 mmol) and Benzyl alcohol (2 mmol) | CNS@Zn_1_-AA (15 mg) | Toluene (2 mL)/Ar atm | KOH (0.3 equiv.) | 120 | 12 | 99% (92%) | 100.8 | 8.4 | ^[14]^ |
| 5 | Aniline (0.055 mmol) and Benzyl alcohol (68 mmol) | Pd_1_/CeO_2_(100) (0.023 mg) | Ar atm | - | 140 | 6 | 99% (90%) | 223.7 | 37.28 | ^[15]^ |
| 6 | Aniline (3 mmol) and Benzyl alcohol (6 mmol) | Fe_20_-SA@NSC  (20 mg) | Ar atm | KOH (0.9 mmol) | 135 | 24 | 99% (94%) | 333.6 | 13.9 | ^[16]^ |
| 7 | Aniline (1 mmol) and Benzyl alcohol (1 mmol) | Ir-PPy Nanocatalyst (25 mg) | Toluene (5 mL)/N_2_ atm | *t-*BuOK (2 mmol) | 100 | 24 | 99% (99%) | 376.8 | 15.7 | ^[17]^ |
| 8 | Aniline (30 mmol) and Benzyl alcohol (60 mmol) | Ni_9.5_/NC (100 mg) | Toluene (10 ml)/Ar (1 bar) | - | 160 | 24 | 36% (86%) | 57.4 | 2.39 | ^[18]^ |
| 9 | Aniline (3 mmol) and Benzyl alcohol (6 mmol) | Ni_2_Fe_2_@CN (5 mg) | o-Xylene (2 mL)/Ar atm | KOH (1.5 mmol) | 140 | 24 | 99% (99%) | 55.51 | 2.31 | ^[19]^ |
| 10 | Aniline (1 mmol) and Benzyl alcohol (1.3 mmol) | TiO_2_ (10 mol % amine) | Toluene (1 mL) | KOH (1 mmol) | 140 | 24 | --  (99%) | 9.8 | 0.41 | ^[20]^ |
| 11 | Aniline (0.5 mmol) and Benzyl alcohol (1 mmol) | CoN_x_@NC (10 mg) | Toluene (2 mL) | *t*-BuOK (0.5 mmol) | 140 | 24 | 99% (92%) | - | - | ^[21]^ |
| 12 | Aniline (0.5 mmol) and Benzyl alcohol (0.5 mmol) | Ni/Ru@SBA (10 mg) | Toluene (2 mL) | *-* | 120 | 24 | 93% (93%) | 78.32 | 3.26 | ^[22]^ |
| 13 | Aniline (0.5 mmol) and Benzyl alcohol (1 mmol) | Co@NC-800-L1 (15 mg) | Toluene (2 mL) | *t*-BuOK (1 equiv.) | 140 | 24 | 99% (99%) | 115.2 | 4.8 | ^[23]^ |
| 14 | Aniline (0.5 mmol) and Benzyl alcohol (0.75 mmol) | Mn@NrGO (8 mol%) | n-Octane (2 mL) | *t*-BuOK (1 mmol) | 140 | 24 | 99% (89%) | - | - | ^[24]^ |
| 15 | Aniline (3 mmol) and Benzyl alcohol (6 mmol) | Fe_10_Pd_1_/NC500 (20 mg) | N_2_ atm | *-* | 120 | 16 | 99% (94%) | 480 | 30.6 | ^[25]^ |
| 16 | Aniline (0.6 mmol) and Benzyl alcohol (0.6 mmol) | Hf-MOF-808_H_2_O (12 mol % Hf) | o-Xylene (1.35 mL) | *-* | 120 | 3 | 97% (90%) | 3.03 | 1.52 | ^[26]^ |
| 17 | Aniline (0.8 mmol) and Benzyl alcohol (0.8 mmol | Hf-Beta Zeolite (50:1 aniline/metal molar ratio) | o-Xylene (1.8 mL) | *-* | 150 | 4.5 | 95% (97%) | 48.6 | 10.8 | ^[27]^ |
| 18 | Aniline (4.61 mmol) and Benzyl alcohol (21.5 mmol) | TiOH-80 (100 mg) | o-Xylene (2 g)/N_2_ atm | *-* | 180 | 15 | 99%  (99%) | 3 | 0.2 | ^[28]^ |
| 19 | Aniline (0.5 mmol) and Benzyl alcohol (1 mmol) | Pd@SiO_2_ (1 mol% Pd) | o-Xylene (1 mL) | *-* | 150 | 30 | 99% (97%) | 96 | 3.2 | ^[29]^ |
| 20 | Aniline (1.38 mmol) and Benzyl alcohol (0.9 mmol) | Nano-Fe_2_O_3_ (30 mmol%) | Toluene (1 mL)/Ar atm | KOH (0.27 mmol) | 135 | 24 | 89% (83%) | 9.6 | 0.4 | ^[30]^ |
| 21 | Aniline (1 mmol) and Benzyl alcohol (1.3 mmol) | Ru/N-C (20 mg) | Toluene (3 mL)/N_2_ atm | KOH (0.5 mmol) | 110 | 24 | 96% (90%) | 451.2 | 18.8 | ^[31]^ |
| 22 | Aniline (0.5 mmol) and Benzyl alcohol (0.7 mL) | Co_2_Rh_2_/C (5 mol %, 45 mg) | N_2_ atm | - | 100 | 24 | -- (90%) | 19.2 | 0.8 | ^[32]^ |

**Table S2.** Analysis of elemental composition of Fe_NP_@N-G and Fe_SA_@N-G by XPS and ICP-MS.

| **Catalyst** | **Element** | **(at.%)^a^** | **B.E. (eV)** | **Catalyst** | **Element** | **(at.%)^a^** | **B.E. (eV)** |
| --- | --- | --- | --- | --- | --- | --- | --- |
| Fe_NP_@N-G | Fe | 1.87 | 712.51 | Fe_SA_@N-G | Fe | 0.95 | 713.26 |
|  | C | 78.29 | 285.04 |  | C | 96.25 | 285.54 |
|  | N | 4.54 | 400.15 |  | N | 2.8 | 400.82 |
|  | Fe | 1.7wt%^b^ | |  | Fe | 1.06wt%^b^ | |

^a^X-ray photoelectron spectroscopy (XPS); ^b^Inductively coupled plasma mass spectrometry (ICP-MS).

**Table S3.** XPS deconvlution results of N 1s and C 1s of Fe_NP_@N-G and Fe_SA_@N-G.

| **N 1s (Fe_NP_@N-G)** | **B. E. (eV)** | **Content (%)** | **N 1s (Fe_SA_@N-G)** | **B. E. (eV)** | **Content (%)** |
| --- | --- | --- | --- | --- | --- |
| **Pyridinic-N** | **398.32** | **21.08** | **Pyridinic-N** | **397.37** | **11.57** |
| **Fe-N_x_** | **399.88** | **24.18** | **Fe-N_x_** | **398.58** | **22.92** |
| **Pyrrolic-N** | **400.95** | **38.86** | **Pyrrolic-N** | **400.05** | **35.49** |
| Graphitic-N | 402.07 | 9.80 | Graphitic-N | 401.29 | 24.93 |
| Oxidized-N | 403.10 | 6.08 | Oxidized-N | 403.06 | 5.09 |
| **C 1s (Fe_NP_@N-G)** | **B. E. (eV)** | **Content (%)** | **C 1s (Fe_SA_@N-G)** | **B. E. (eV)** | **Content (%)** |
| C=C | 284.79 | 64.93 | C=C | 284.84 | 81.70 |
| C-N/C-O | 285.90 | 6.15 | C-N/C-O | 286.79 | 4.75 |
| C=N/C=O | 286.47 | 9.76 | C=N/C=O | 288.14 | 8.26 |
| C-C=O/N-C=O | 287.42 | 19.16 | C-C=O/N-C=O | 289.75 | 5.29 |

**Table S4.** The fitting data of EXAFS

| **Sample** | **Pre-edge Energy, keV** | **XANES Energy, keV** | **CN** | **R, Å** | **Δσ^2^**  **(x 10^3^)** | **Eo, (eV)** |
| --- | --- | --- | --- | --- | --- | --- |
| Fe Foil | - | 7.1120 | - | - | - | - |
| FeO | 7.1126 | 7.1190 | - | - | - | - |
| Fe_2_O_3_ | 7.1144 | 7.1233 | - | - | - | - |
| Fe_SA_@N-G | 7.1144 | 7.1128 | 3.8 | 2.00 | 0.009 | -0.9 |

CN: Coordination number; R: Bonding distance; σ^2^ : Debye Waller factor; Eo: Inner potential shift

**Table S5.** Porosity properties of samples.

| **Sample** | **^aS^BET (m^2^ g^-1^)** | **Pore volume (cm^3^ g^-1^)** | **Average pore size (nm)** |
| --- | --- | --- | --- |
| N-G | 944 | 216.87 | 0.92 |
| Fe_SA_@N-G | 788 | 181.08 | 0.61 |
| Fe_NP_@N-G | 614 | 141.04 | 0.58 |

**
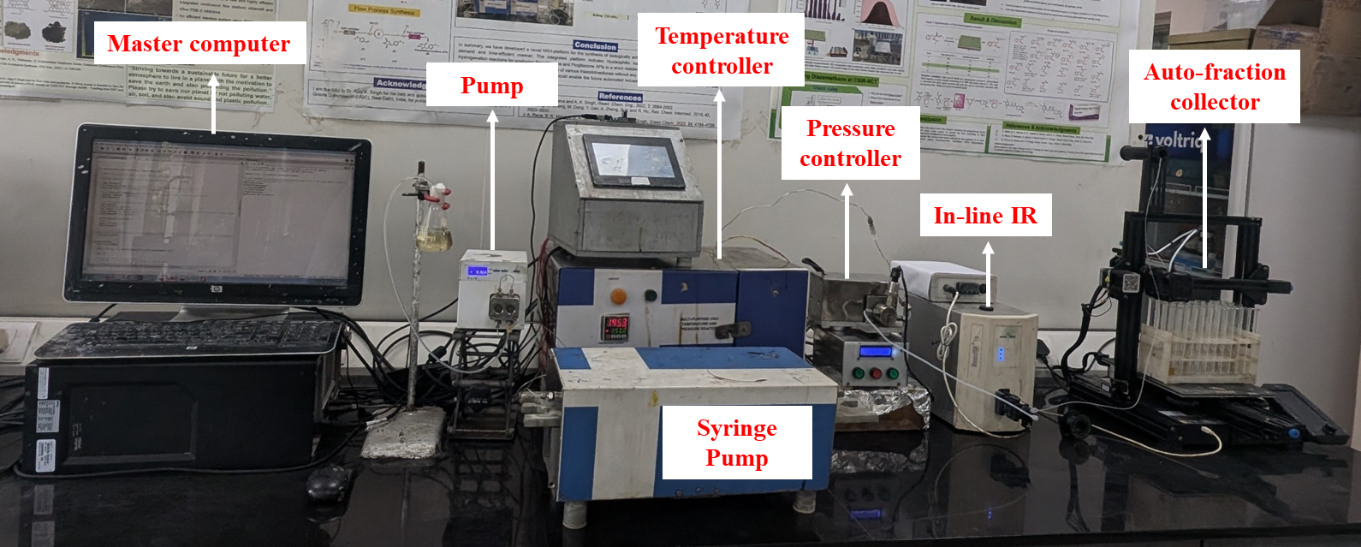
**

**Figure S7.** Actual picture of continuous flow setup integrated with artificial intelligence (AI).

The HPLC pumps (**Figure S7**) were directly connected to the main computer using either an RS-232 interface or a LAN. These pumps are designed to introduce reactants and reagent solutions into the micro reactor at variable flow rates (fr), as specified by the central computer. The control protocol allows for starting and stopping the pumps as needed. Communication between the central computer and the HPLC pumps was established through serial communication, utilizing ASCII code to exchange key information, such as flow rate, operational status, and duration. This setup ensured seamless transmission of essential data from the main computer to the pumps, enabling their operation as required.

**Table S6:** Optimization of continuous-flow coupling of aniline with benzyl alcohol on Fe_SA_@N-G catalyst.

**
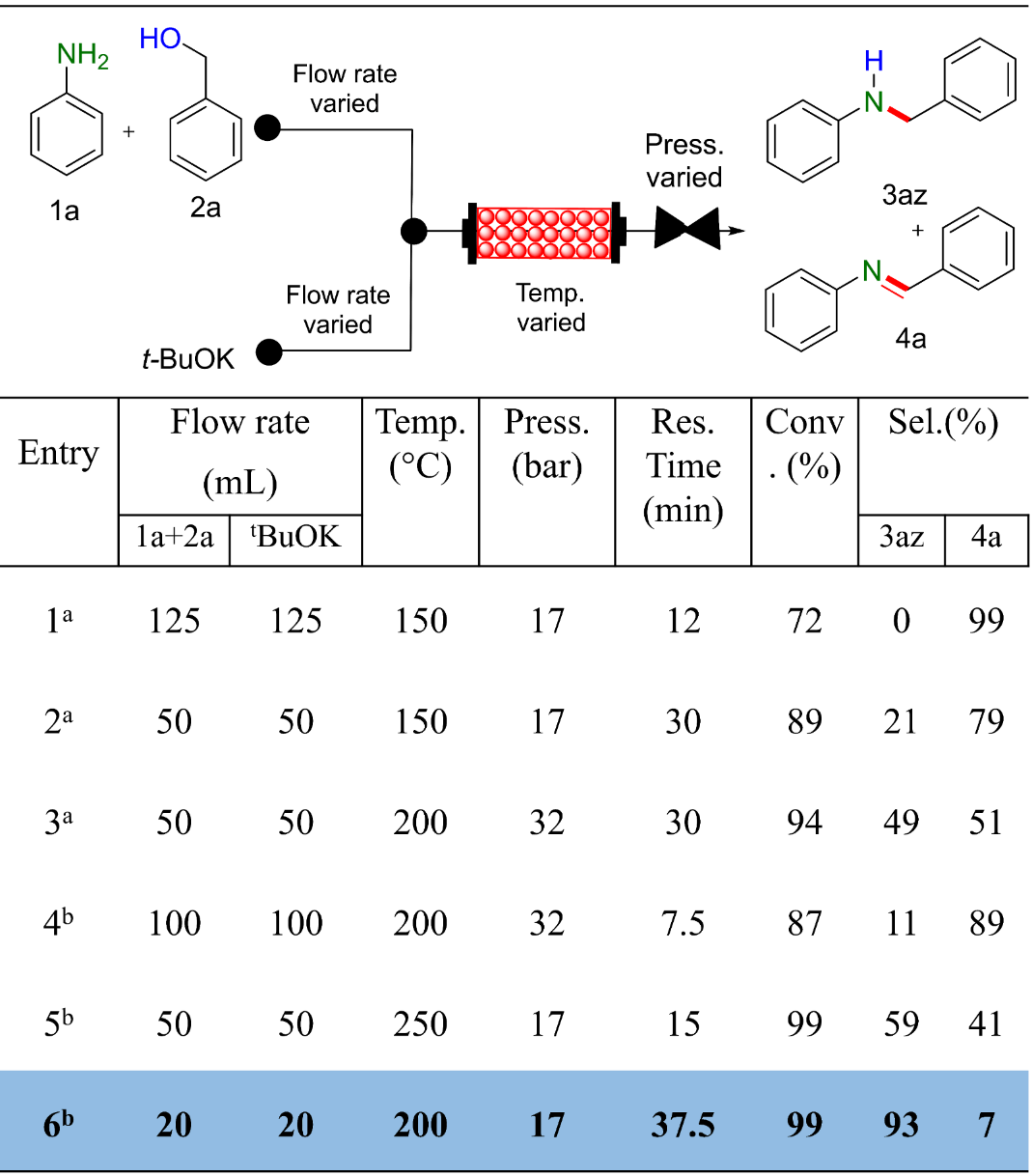
**

**Reaction conditions:** a) 0.1 M of 1a + 0.5 eq. of *t*-BuOK in toluene; 0.3 M solution of 2a in toluene; 7 mL cartridge filled with 600 mg of catalyst and silica with 3 mL volume free space; b) 0.1 M of 1a in toluene +: benzyl alcohol (1:4); 0.05 M of *t*-BuOK in toluene; 7 mL cartridge filled with 1 g of catalyst and silica with 1.5 mL volume free space.

1. **Density functional theory (DFT) study:**

For understanding the catalytic reaction mechanism and its energetics, periodic density functional theory (DFT) calculations were carried out using the Vienna *ab initio* simulation package (VASP).^33-35^ Interactions between the core and valence electrons were treated through the Projector augmented wave (PAW) potentials.^36^ Exchange-correlation energy density functional of Perdew-Burke-Ernzerhof (PBE)^37^ was used along with Grimme’s D3 semiempirical method (PBE-D3) to treat the dispersion interactions.^38^ Electronic wave functions of the valence electrons were expanded using the Plane-wave basis-sets with a kinetic energy cutoff of 550 eV. A single layer graphene with 8 x 8 x 1 supercell consisting of 128 carbon atoms was considered to generate the model systems. Vacuum layer of 20 Å was considered along the non-periodic direction (perpendicular to the graphene layer) to avoid image-image interactions. Position of all the atoms and unit cell were relaxed under constant volume constraint with a force cutoff of 0.005 eV/Å. All the reported structures were generated using Chimera^39^ and BKChem^40^ packages. Binding energies (ΔE) of different intermediates with the surface were measured as

ΔE (x) = E(s-x)-[E(s)+E(x)]

where E(s-x), E(s) and E(x) represent energies of adsorbate(x) bound surface, free surface and adsorbate respectively. Energies of all the molecular systems were calculated by optimizing the molecular structure in a large cubic cell of 20 Å lengths. Energy of Fe metal atoms was considered as the cohesive energy per Fe atom calculated from the bulk Fe structure.

**
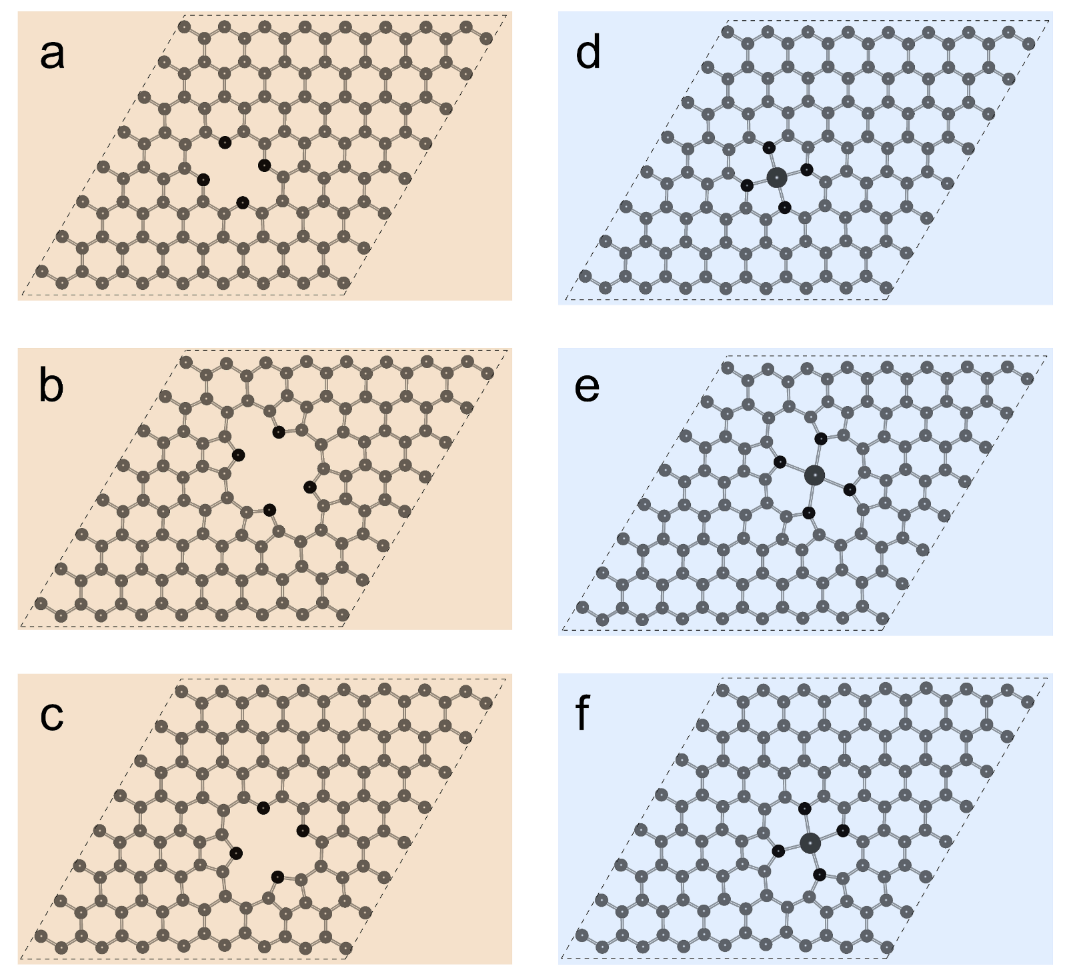
**

**Figure S8.**  a-c) Three different types of N_4_ units incorporated in graphene along with their d-f) Fe bound counterparts.

**
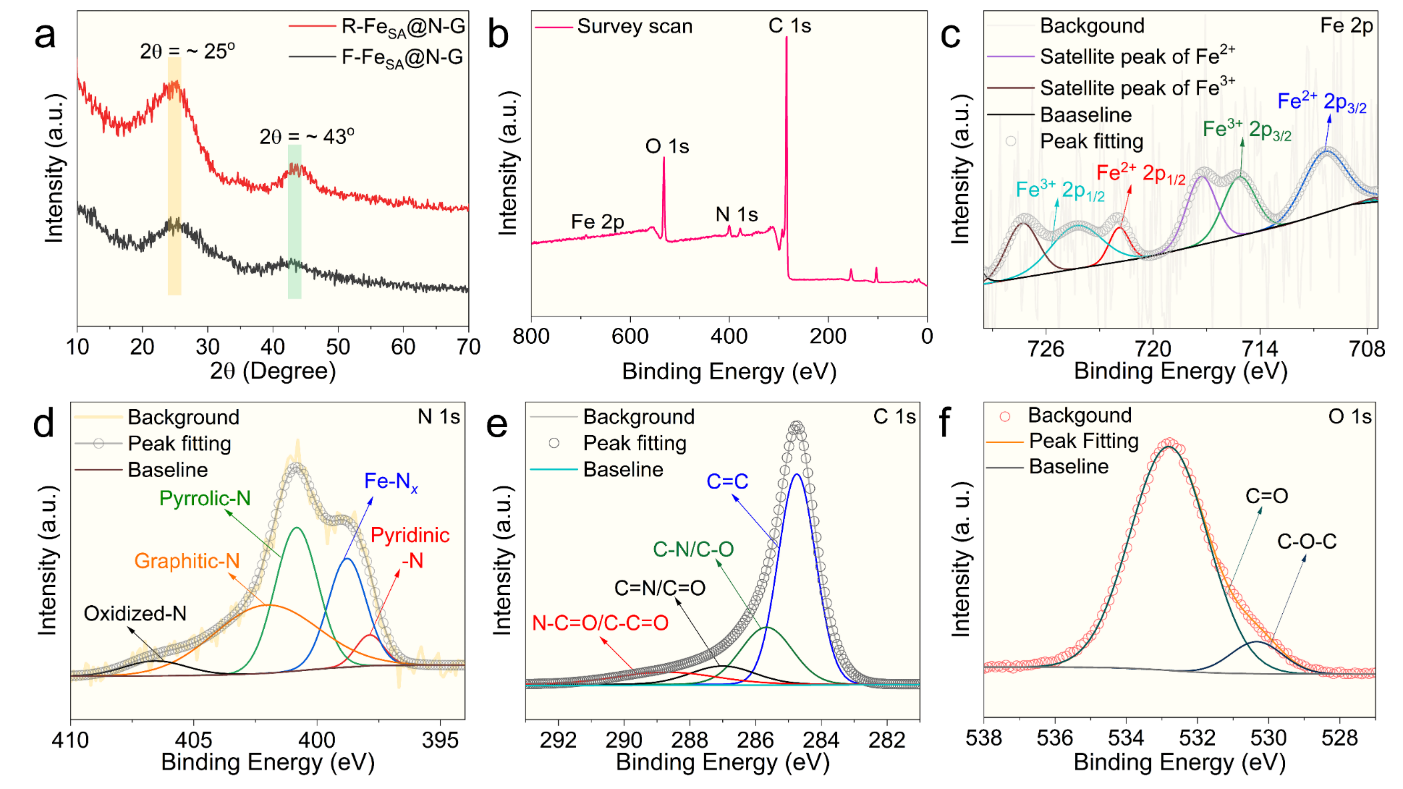
**

**Figure S9.** a) XRD pattern of fresh and reused Fe_SA_@N-G. XPS analysis of reused Fe_SA_@N-G: b) Survey scan, c) deconvolution of Fe 2p, d) deconvolution of N 1s, e) deconvolution of C 1s, and deconvolution of O 1s.

1. **^1^H- and ^13^C-NMR Spectra**

**^1^H- and ^13^C-NMR spectra of N-benzylaniline (3az)**

The reaction mixture was extracted and separated through regular batch process. The organic phase was concentrated under reduced pressure to give the product 3az (0.147 g in 12 h, 90%) as a colorless oil.

**^1^H NMR (400 MHz, CDCl_3_)** δ 7.39 – 7.30 (m, 4H), 7.29 – 7.24 (m, 1H), 7.20 – 7.12 (m, 2H), 6.74 – 6.68 (m, 1.0 Hz, 1H), 6.65 – 6.61 (m, 2H), 4.32 (s, 2H), 4.01 (s, 1H).


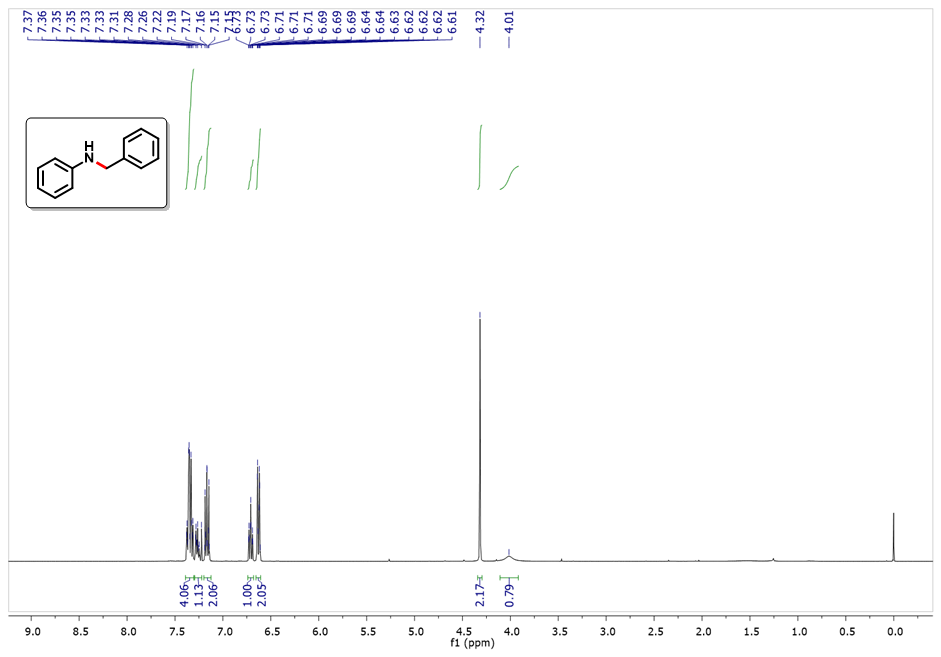


**Figure S10.** ^1^H-NMR (400 MHz, CDCl_3_) spectra of N-benzylaniline (**3az**).

**^13^C NMR (126 MHz, CDCl_3_)** δ 148.19, 139.48, 129.32, 128.69, 127.57, 127.28, 117.63, 112.91, 48.39.


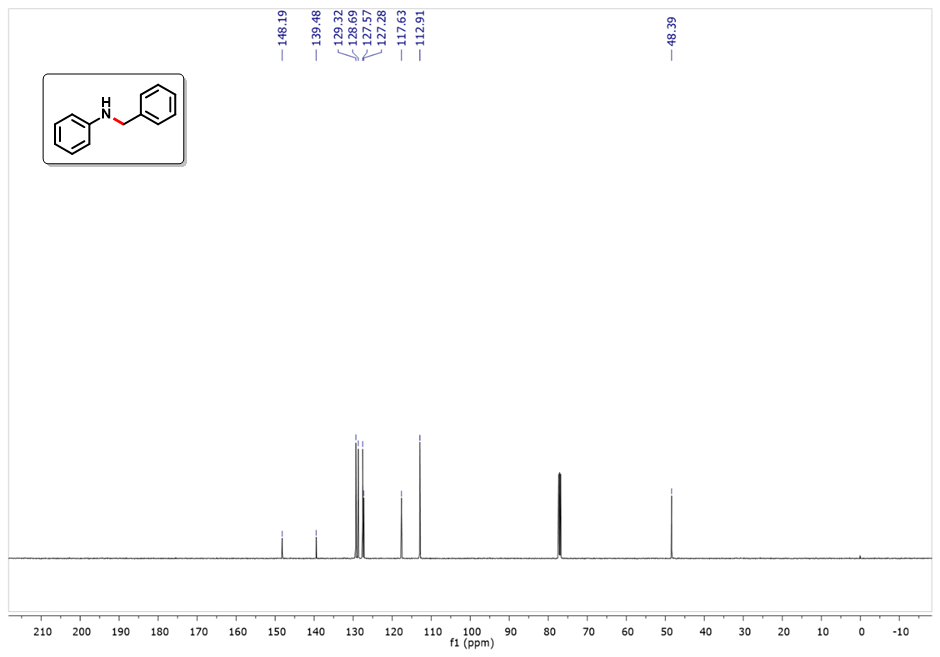


**Figure S11.** ^13^C-NMR (400 MHz, CDCl_3_) spectra of N-benzylaniline (**3az**).

**^1^H- and ^13^C-NMR spectra of N-benzylpyridin-2-amine (3aaa)**

The title compound was synthesised following the general procedure described in experimental section, and involve corresponding reactant exchange with compound (pyridin-2-amine). The crude mixture was concentrated under vacuum and the product was purified by flash chromatography; R*f* = 0.5 (10% ethylacetate/hexane); to give compound 3aaa (0.152 g in 12 h, 92%) as a colorless oil.

**^1^H NMR (400 MHz, CDCl_3_)** δ 8.03 (d, *J* = 4.1 Hz, 1H), 7.34 – 7.18 (m, 6H), 6.53 – 6.50 (m, 1H), 6.30 (d, *J* = 8.4 Hz, 1H), 4.86 (s, 1H), 4.43 (d, *J* = 5.6 Hz, 2H).


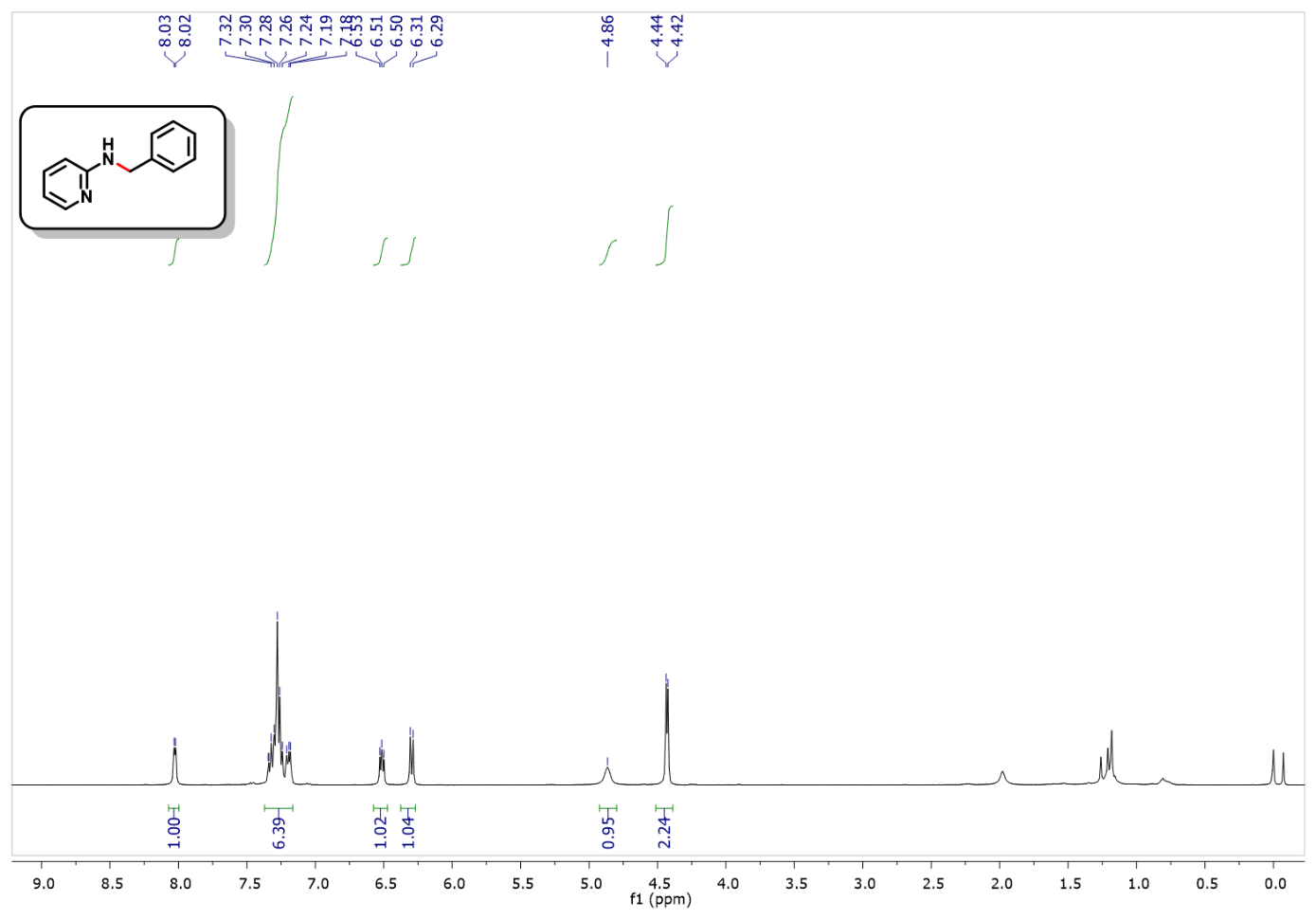


**Figure S12.** ^1^H-NMR (400 MHz, CDCl_3_) spectra of N-benzylpyridin-2-amine (**3aaa**).

**^13^C NMR (126 MHz, CDCl_3_)** δ 157.58, 147.07, 138.12, 136.51, 127.61, 126.36, 126.21, 112.12, 105.79, 45.30.

**
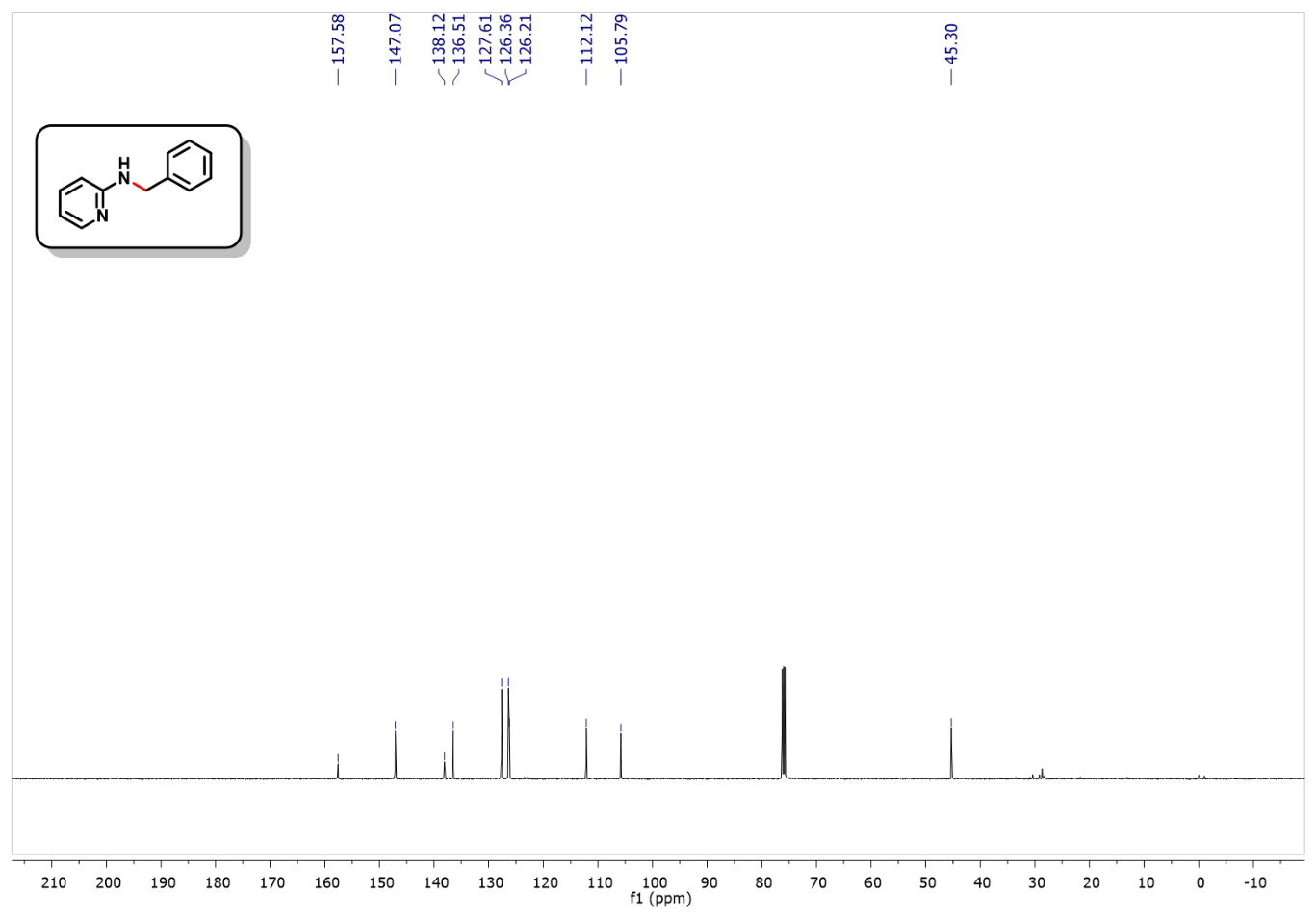
**

**Figure S13.** ^13^C-NMR (126 MHz, CDCl_3_) spectra of N-benzylpyridin-2-amine (**3aaa**).

**^1^H- and ^13^C-NMR spectra of N-benzylnaphthalen-1-amine (3aab)**

The title compound was synthesised following the general procedure described in experimental section, and involve corresponding reactant exchange with compound 1c (naphthalen-1-amine). The crude mixture was concentrated under vacuum and the product was purified by flash chromatography; R*f* = 0.5 (10% ethylacetate/hexane); to give compound 3aab (0.167 g in 12 h, 80%) as a colorless oil.

**^1^H NMR (400 MHz, CDCl_3_)** δ 7.81 – 7.78 (m, 2H), 7.46 – 7.21 (m, 9H), 6.62 – 6.60 (m, 1H), 4.69 (s, 1H), 4.47 (s, 2H).


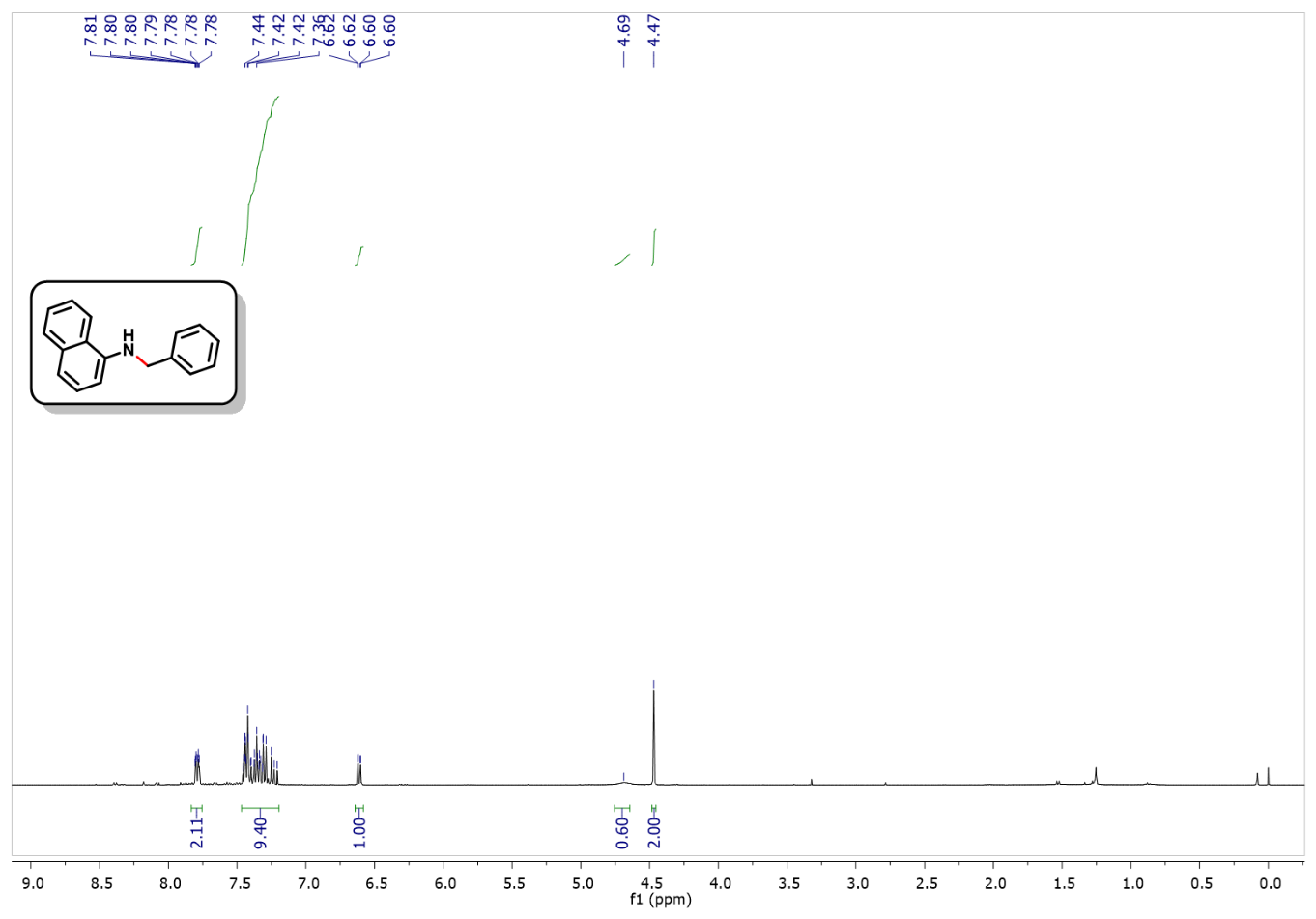


**Figure S14.** ^1^H-NMR (400 MHz, CDCl_3_) spectra of N-benzylnaphthalen-1-amine (**3aab**).

**^13^C NMR (101 MHz, CDCl_3_)** δ 143.16, 139.08, 134.35, 129.05, 128.99, 128.89, 128.78, 128.59, 127.83, 127.48, 127.20, 126.66, 125.82, 124.84, 123.46, 119.97, 117.80, 104.96, 48.73.


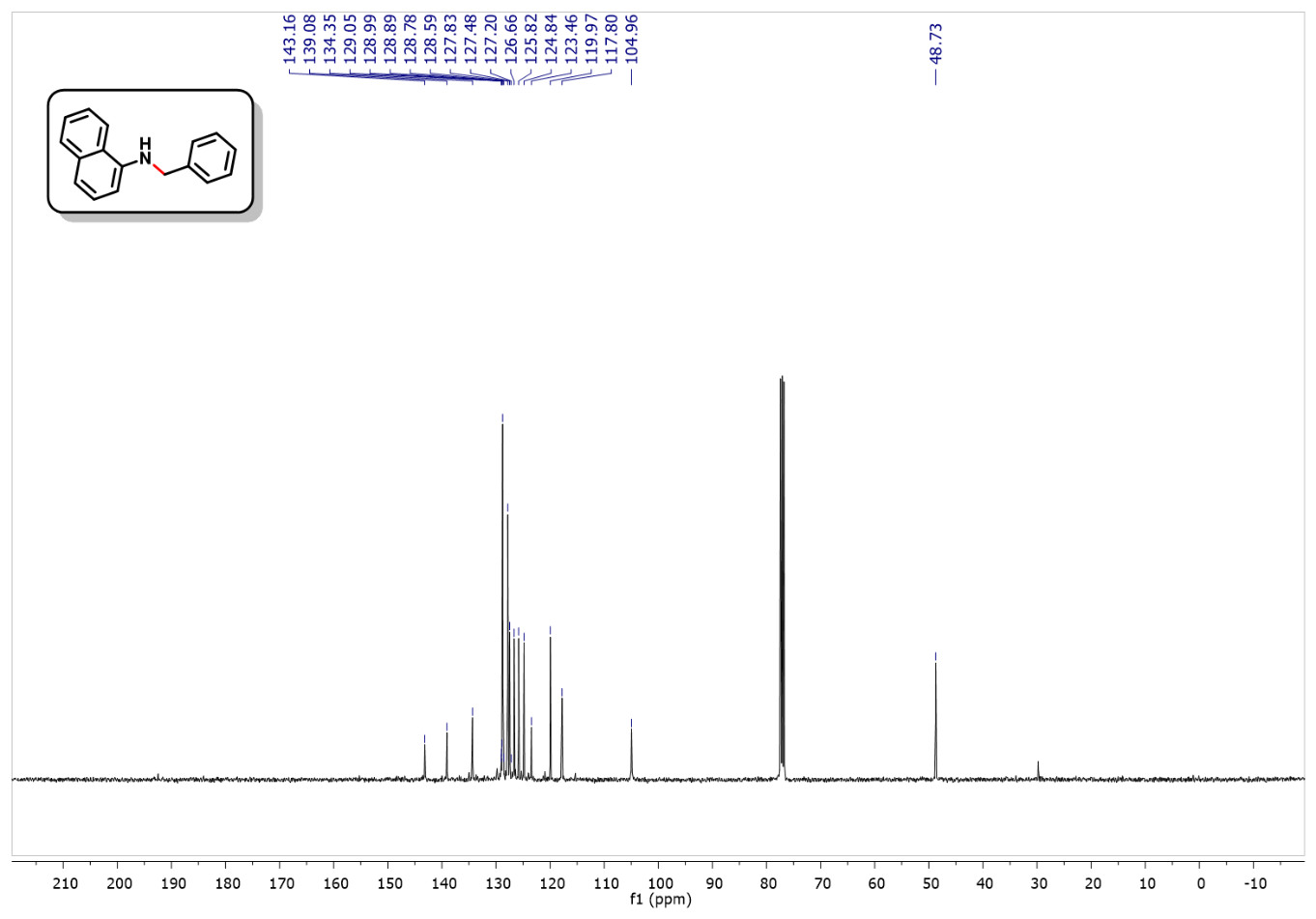


**Figure S15.** ^13^C-NMR (101 MHz, CDCl_3_) spectra of N-benzylnaphthalen-1-amine (**3aab**).

**^1^H- and ^13^C-NMR spectra of N-benzyl-3,4,5-trimethoxyaniline (3aac)**

The title compound was synthesised following the general procedure described in experimental section, and involve corresponding reactant exchange with compound 1c (naphthalen-1-amine). The crude mixture was concentrated under vacuum and the product was purified by flash chromatography; R*f* = 0.6 (10% ethylacetate/hexane); to give compound 3aac (0.212 g in 12 h, 87%) as a white solid. **mp:** 91-93 °C,

**^1^H NMR (400 MHz, CDCl_3_)** δ 7.26 – 7.20 (m, 4H), 7.17 – 7.13 (m, 1H), 5.74 (s, 2H), 4.16 (s, 2H), 3.64 (s, 3H), 3.63 (s, 6H).


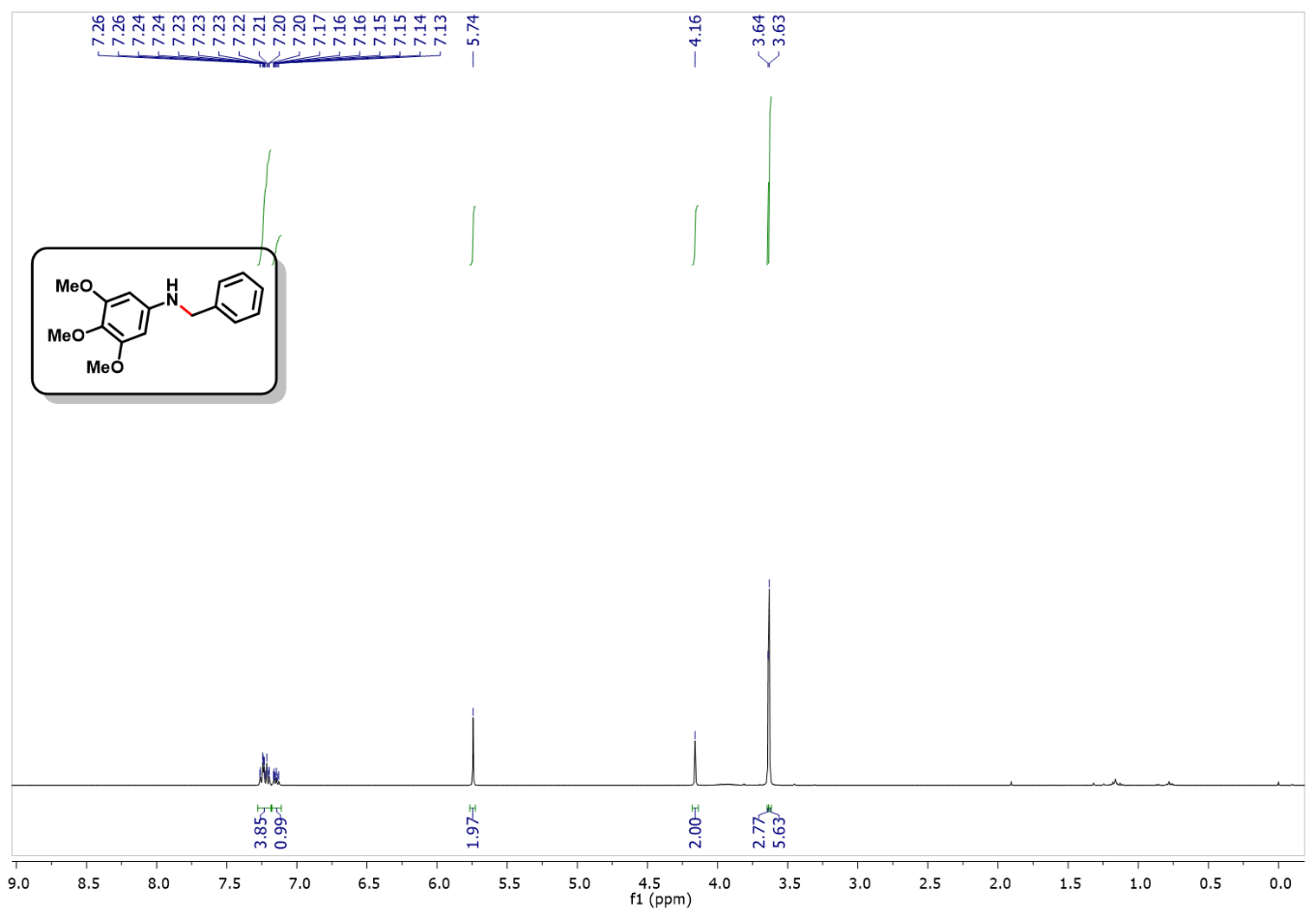


**Figure S16.** ^1^H-NMR (400 MHz, CDCl_3_) spectra of N-benzyl-3,4,5-trimethoxyaniline (**3aac**).

**^13^C NMR (75 MHz, CDCl_3_)** δ 153.96, 145.17, 139.54, 130.07, 128.68, 127.56, 127.30, 90.52, 61.09, 55.89, 48.81.


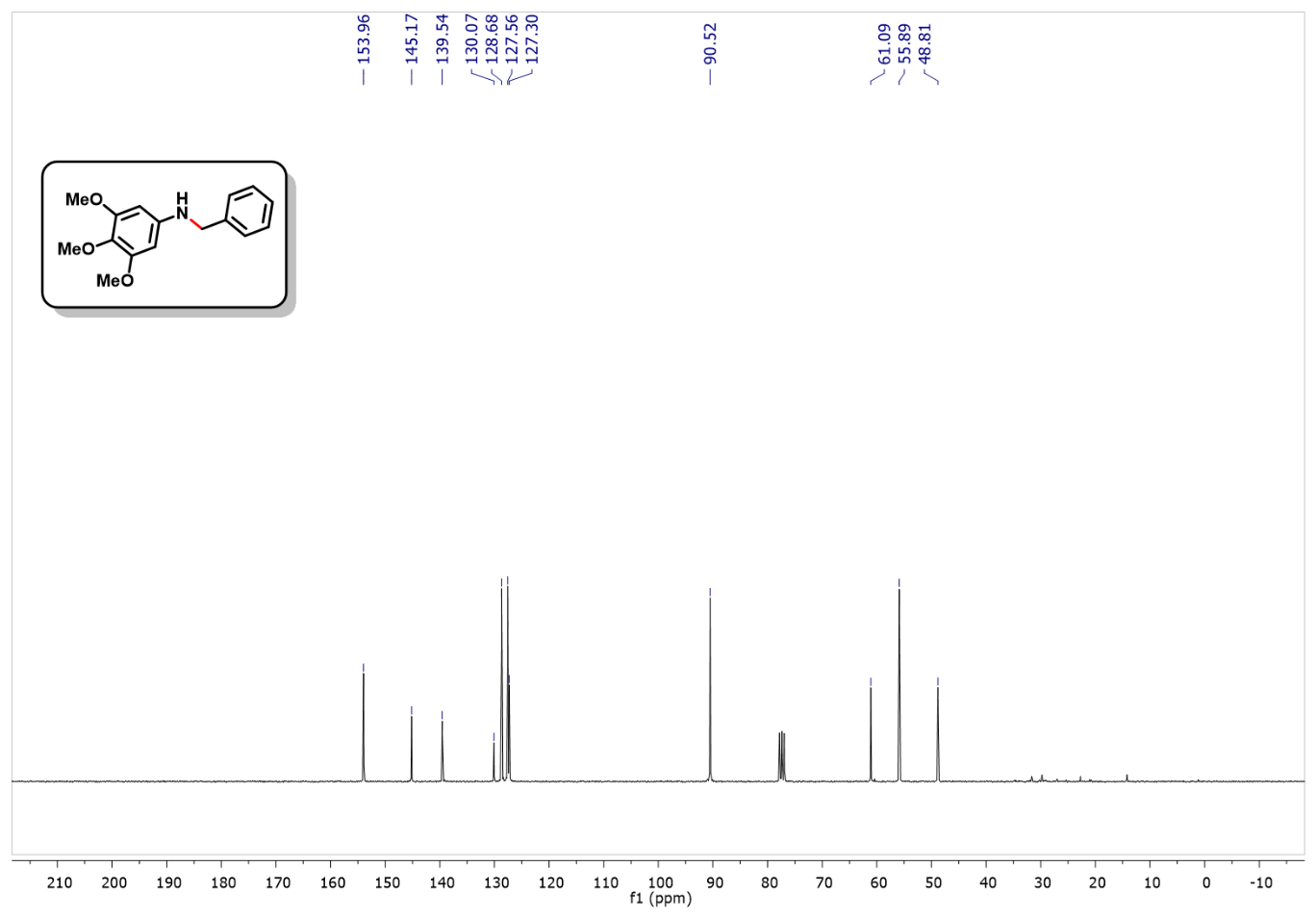


**Figure S17.** ^13^C-NMR (75 MHz, CDCl_3_) spectra of N-benzyl-3,4,5-trimethoxyaniline (**3aac**).

1. **References**

[1] A. Bakandritsos, R.G. Kadam, P. Kumar, G. Zoppellaro, M. Medved, J. Tucek, T. Montini, O. Tomanec, P. Andryskova, B. Drahos, R.S. Varma, M. Otyepka, M.B. Gawande, P. Fornasiero, R. Zboril, *Adv. Mater*. **2019**, 31, e1900323.

[2] K. Sun, H. Shan, H. Neumann, G.P. Lu, M. Beller, *Nat. Commun*. **2022**, 13, 1848.

[3] T. Sharifi, E. Gracia-Espino, H.R. Barzegar, X. Jia, F. Nitze, G. Hu, P. Nordblad, C.W. Tai, T. Wagberg, *Nat. Commun*. **2013**, *4*, 2319.

[4] Z. Guo, C. Li, Y. Li, Y. Wen, Y. Wu, B. Jia, K. Tai, X. Zeng, X. Li, J. Liu, Z. Ouyang, *Nat. Commun*. **2022**, *13*, 7177.

[5] A.M. Agour, E. Elkersh, G.E. Khedr, H.G. El-Aqapa, N.K. Allam, *ACS Appl. Nano Mater.* **2023**, *6*, 15980-15989.

[6] L. Jiao, G. Wan, R. Zhang, H. Zhou, S.H. Yu, H.L. Jiang, *Angew. Chem. Int. Ed. Engl.* **2018**, *57*, 8525-8529.

[7] T. Varga, L. Vasarhelyi, G. Ballai, H. Haspel, A. Oszko, A. Kukovecz, Z. Konya, *ACS Omega* **2019**, *4*, 130-139.

[8] Q. Dong, X. Zhuang, Z. Li, B. Li, B. Fang, C. Yang, H. Xie, F. Zhang, X. Feng, *J. Mater. Chem. A* **2015**, *3*, 7767-7772.

[9] Y. Wu, Y. Huang, X. Dai, F. Shi, *ChemSusChem* **2019**, *12*, 3185-3191.

[10] S.N. Bhange, S.M. Unni, S. Kurungot, *ACS Appl. Energy Mater.* **2018**, *1*, 368-376.

[11] Y. Chen, S. Ji, Y. Wang, J. Dong, W. Chen, Z. Li, R. Shen, L. Zheng, Z. Zhuang, D. Wang, Y. Li, *Angew. Chem. Int. Ed. Engl.* **2017**, *56*, 6937-6941.

[12] W. Chen, M. Sohail, Y. Veeranna, Y. Yang, A. A. Bengali, H.-C. Zhou, S. T. Madrahimov, *ACS Appl. Mater. Interfaces* **2025**, *17*, 12, 17775–17782.

[13] X. Liu, L. Huang, Y. Ma, G. She, P. Zhou, L. Zhu, Z. Zhang, *Nat. Commun*. **2024**, *15*, 7012.

[14] X. Zhang, Q. Zhang, J. Reng, Y. Lin, Y. Tang, G. Liu, P. Wang, G.P. Lu, *Nanomaterials* **2023**, *13*, 445.

[15] B. Hu, K. Sun, Z. Zhuang, Z. Chen, S. Liu, W.C. Cheong, C. Chen, M. Hu, X. Cao, J. Ma, R. Tu, X. Zheng, H. Xiao, X. Chen, Y. Cui, Q. Peng, C. Chen, Y. Li, *Adv. Mater.* **2022**, *34*, e2107721.

[16] G.-P. Lu, H. Shan, Y. Lin, K. Zhang, B. Zhou, Q. Zhong, P. Wang, *J. Mater. Chem. A* **2021**, *9*, 25128-25135.

[17] M. Iuliano, M. Sarno, C. Cirillo, E. Ponticorvo, S. De Pasquale, *Europ. J. Inorg. Chem.* **2021**, *2021*, 644-653.

[18] H. Su, P. Gao, M.Y. Wang, G.Y. Zhai, J.J. Zhang, T.J. Zhao, J. Su, M. Antonietti, X.H. Li, J.S. Chen, *Angew. Chem. Int. Ed. Engl.* **2018**, *57*, 15194-15198.

[19] G. Wang, L. Sun, W. Liu, H. Zhan, S. Bi, *Nano Res.* **2023**, 17, 2308–2319.

[20] R. Upadhyay, S.K. Maurya, *J. Org. Chem.* **2023**, *88*, 16960-16966.

[21] V. Vyas, P. Maurya, A. Indra, *Chem. Sci.* **2023**, *14*, 12339-12344.

[22] L. Wang, X. Jv, R. Wang, L. Ma, J. Liu, J. Sun, T. Shi, L. Zhao, X. Zhang, B. Wang, *ACS Sustain. Chem. Eng.* **2022**, *10*, 8342-8349.

[23] Z. Ma, B. Zhou, X. Li, R.G. Kadam, M.B. Gawande, M. Petr, R. Zboril, M. Beller, R.V. Jagadeesh, *Chem. Sci.* **2021**, *13*, 111-117.

[24] M. Subaramanian, P.M. Ramar, G. Sivakumar, R.G. Kadam, M. Petr, R. Zboril, M.B. Gawande, E. Balaraman, *ChemCatChem* **2021**, *13*, 4334-4341.

[25] P.-y. Wu, G.-p. Lu, C. Cai, *Green Chem.* **2021**, *23*, 396-404.

[26] B. Bohigues, S. Rojas-Buzo, M. Moliner, A. Corma, *ACS Sustain. Chem. Eng.* **2021**, *9*, 15793-15806.

[27] S. Rojas-Buzo, P. Concepción, A. Corma, M. Moliner, M. Boronat, *ACS Catal*. **2021**, *11*, 8049-8061.

[28] F. Niu, Q. Wang, Z. Yan, B.T. Kusema, A.Y. Khodakov, V.V. Ordomsky, *ACS Catal.* **2020**, *10*, 3404-3414.

[29] A.S. Alshammari, K. Natte, N.V. Kalevaru, A. Bagabas, R.V. Jagadeesh, *J. Catal*. **2020**, *382*, 141-149.

[30] M. Nallagangula, C. Sujatha, V.T. Bhat, K. Namitharan, *Chem. Commun.* **2019,** *55*, 8490-8493.

[31] B. Guo, H.X. Li, S.Q. Zhang, D.J. Young, J.P. Lang, *ChemCatChem* **2018**, *10*, 5627-5636.

[32] H. Chung, Y.K. Chung, *J. Org. Chem.* **2018**, *83*, 8533-8542.

[33] G. Kresse, J. Furthmüller, *Phys. Rev. B* **1996**, *54*, 11169–11186.

[34] G. Kresse, J. Furthmüller, *Comput. Mater. Sci.* **1996**, *6*, 15–50.

[35] G. Kresse, D. Joubert, *Phys. Rev. B* **1999**, *59*, 1758–1775.

[36] P. E. Blöchl, *Phys. Rev. B* **1994**, *50*, 17953–17979.

[37] J. P. Perdew,K. Burke, M. Ernzerhof, *Phys. Rev. Lett.* **1996**, *77*, 3865–3868.

[38] S. Grimme, S. Ehrlich, L. Goerigk, *J. Comput. Chem.* **2011**, *32*, 1456–1465.

[39] E. F. Pettersen, T. D. Goddard, C. C. Huang, G. S. Couch, D. M. Greenblatt, E. C. Meng, T. E. Ferrin, *J. Comput. Chem.* **2004**, *25*, 1605-12.

[40] https://bkchem.zirael.org/
